# Whole genome resequencing identifies candidate genes and allelic diagnostic markers for resistance to *Ralstonia solanacearum* infection in cultivated peanut (*Arachis hypogaea* L.)

**DOI:** 10.3389/fpls.2022.1048168

**Published:** 2023-01-04

**Authors:** Chong Zhang, Wenping Xie, Huiwen Fu, Yuting Chen, Hua Chen, Tiecheng Cai, Qiang Yang, Yuhui Zhuang, Xin Zhong, Kun Chen, Meijia Gao, Fengzhen Liu, Yongshan Wan, Manish K. Pandey, Rajeev K. Varshney, Weijian Zhuang

**Affiliations:** ^1^ Key Laboratory of Ministry of Education for Genetics, Breeding and Multiple Utilization of Crops, College of Agriculture, Institute of Oil Crops Research, Research Center for Genetics and Systems Biology of Leguminous Oil Plants, Fujian Agriculture and Forestry University, Fuzhou, China; ^2^ State Key Laboratory of Ecological Pest Control for Fujian and Taiwan Crops, Fujian Agriculture and Forestry University, Fuzhou, China; ^3^ State Key Laboratory of Crop Biology, Shandong Key Laboratory of Crop Biology, College of Agronomy, Shandong Agricultural University, Tai’an, China; ^4^ Center of Excellence in Genomics and Systems Biology (CEGSB), International Crops Research Institute for the Semi-Arid Tropics (ICRISAT), Hyderabad, India; ^5^ Murdoch’s Centre for Crop and Food Innovation, State Agricultural Biotechnology Centre, Food Futures Institute, Murdoch University, Murdoch, WA, Australia

**Keywords:** peanut, resistance to *Ralstonia solanacearum*, QTL-seq analysis, candidate genes, diagnostic markers

## Abstract

Bacterial wilt disease (BWD), caused by *Ralstonia solanacearum* is a major challenge for peanut production in China and significantly affects global peanut field productivity. It is imperative to identify genetic loci and putative genes controlling resistance to *R. solanacearum* (RRS). Therefore, a sequencing-based trait mapping approach termed “QTL-seq” was applied to a recombination inbred line population of 581 individuals from the cross of Yueyou 92 (resistant) and Xinhuixiaoli (susceptible). A total of 381,642 homozygous single nucleotide polymorphisms (SNPs) and 98,918 InDels were identified through whole genome resequencing of resistant and susceptible parents for RRS. Using QTL-seq analysis, a candidate genomic region comprising of 7.2 Mb (1.8–9.0 Mb) was identified on chromosome 12 which was found to be significantly associated with RRS based on combined Euclidean Distance (ED) and SNP-index methods. This candidate genomic region had 180 nonsynonymous SNPs and 14 InDels that affected 75 and 11 putative candidate genes, respectively. Finally, eight nucleotide binding site leucine rich repeat (NBS-LRR) putative resistant genes were identified as the important candidate genes with high confidence. Two diagnostic SNP markers were validated and revealed high phenotypic variation in the different resistant and susceptible RIL lines. These findings advocate the expediency of the QTL-seq approach for precise and rapid identification of candidate genomic regions, and the development of diagnostic markers that are applicable in breeding disease-resistant peanut varieties.

## 1 Introduction

Bacterial wilt that is caused by *Ralstonia solanacearum* (*R. solanacearum*), is the most damaging bacterial disease that globally affects over 50 and 450 botanical families and plant species, respectively, including several economically important crops such as tobacco, peanut, tomato, and pepper (Salanoubat et al., 2002; Zhang et al., 2017). *R. solanacearum* is a free-living saprophyte that endures in soil and aquatic habitats for long durations (Genin and Boucher, 2004). *R. solanacearum* mostly infects plant roots, propagates in the xylem, disseminates into the stem, and then to the entire plant resulting in wilt and eventual death (Schell, 2000). Bacterial wilt disease often significantly reduces, by 10~30%, the yield and quality of peanut and other important crops; it may also result in complete yield loss (Zhang et al., 2017). Currently, no effective pesticide and biological control method exists to control this pathogen because of its wide host range and durable survival ability (Yu et al., 2011). Nevertheless, cultivating crop varieties that are genetically resistant has efficiently controlled this disease (Sunkara et al., 2014; Reddy, 2016), leading to the development and release of many resistant varieties of the peanut. However, there exists a looming threat of a breakdown of this genetic resistance in China, due to similar resistance mechanisms in both the cultivated varieties (such as Xiekangqing, Taishan Zhenzhu) and wild species (Janila et al., 2016; Luo et al., 2020). Despite the current search for varieties whose resistance is conferred *via* alternate mechanisms, it is imperative to determine the genomic regions and genes that encode resistance to augment the development of new varieties *via* genomics-assisted breeding (GAB) (Pandey et al., 2020).

Through analysis of various plant genomes, map-based cloning of plant genes that confer resistance to *R. solanacearum* was conducted for a few crop species. In *Arabidopsis*, a recessive *RRS1-R* encoding a Tir-NBS-LRR resistant protein with a WRKY domain in resistant line Nd-1 was first identified and cloned by fine mapping (Deslandes et al., 2003). It conferred resistance to GMI1000 when transferred into Col-5 variants with the dominant susceptible allele. Furthermore, another resistant gene *RPS4* was in the reverse orientation and directly upstream of *RRS1-R.* This physical association triggered host resistance to the pathogen (Narusaka et al., 2009). Relatedly, RRS1-R associated with RPS4 is a dimer that recognizes PopP2 of *R. solanacearum* to trigger RRS (Narusaka et al., 2014). In *Arabidopsis*, three quantitative trait loci (QTLs) for RRS were identified in 100 F_9_ recombinant inbred lines (RILs) from another cross of Col-0 × L*er* (Godiard et al., 2003). A putative leucine-rich repeat receptor-like kinase (LRR-RLK) gene named *ERECTA* was cloned and found to trigger RRS (Godiard et al., 2003). Recently, in peanut, two genes *AhRRS5* and *AhRLK1* (also known as *AhCLAVATA1*), encoding an NBS-LRR resistance protein and a receptor-like protein kinase, respectively, were identified by reverse genetics. Transgenic tobaccos that overexpressed these two genes conferred a significantly increased level of resistance to RRS, indicating that both *R* genes and RLKs are involved in resistance mechanisms against BWD (Zhang et al., 2017; Zhang et al., 2019). Hitherto, no resistance genes from other plants have been cloned and characterized by the map-based method.

Recently, several QTLs associated with RRS were effectively identified by QTL mapping in many crop species, including tomato (Thoquet et al., 1996; Carmeille et al., 2006; Wang et al., 2013; Shin et al., 2020), pepper (Mimura et al., 2009) (Du et al., 2019), potato (Habe et al., 2019), eggplant (Lebeau et al., 2013; Salgon et al., 2017), tobacco (Wang et al., 2013) and *Medicago truncatula* (Ben et al., 2013). Up to now, both sequencing-based trait mapping and gene discovery techniques are highly utilized due to low sequencing costs and the development of new methods that elucidate genomic loci and candidate genes associated with specific traits (Varshney et al., 2019; Pandey et al., 2020). Such efforts facilitate faster development of diagnostic markers which can be employed in GAB to accelerate the development of new peanut varieties (Pandey et al., 2020). In peanut, Zhao et al. (Zhao et al., 2016) first reported mapping QTL for RRS on the B02 chromosome using a moderately dense linkage map of 237 SSR and SNP markers. By combining restriction-site-associated DNA sequencing (RAD-seq) and bulk segregant analysis (BSA) techniques, they developed resistant-related SNP markers from the RIL population of crosses between resistant (Yueyou 92) and susceptible (Xinhuixiaoli) varieties. The two detected QTLs (*qBW-1* and *qBW-2*) in the aforementioned RRS study accounted for 21% and 12% of the resistance phenotypic variance in the F_2_ generation, respectively. Only two side-by-side QTLs were found at the *qBW-1* locus on the B02 chromosome in the F_8_ generation. The resistant resource of Yueyou 92 was from a Chinese landrace Xiekangqing, which is a major source of parental types used for breeding BWD-resistant variants in South China (Janila et al., 2016; Luo et al., 2020).

The rapid QTL-seq approach is critical for identifying genomic regions of a trait of interest in plants and identifies QTLs based on BSA and next-generation sequencing (Takagi et al., 2013). QTL-seq was the preferred choice of a fast and effective method that identifies and maps QTLs of target traits in crop plants (Takagi et al., 2013). For example, it was to identify QTLs of the target trait in rice (Arikit et al., 2019; Bommisetty et al., 2020; Lei et al., 2020; Yang et al., 2021), cucumber (Lu et al., 2014; Cao et al., 2021; Zhang et al., 2021), chickpea (Das et al., 2014; Singh et al., 2016; Srivastava et al., 2017), tomato (Illa et al., 2015; Topcu et al., 2021), oilseed rape (Wang et al., 2016; Tudor et al., 2020; Dong et al., 2021), maize (Chen et al., 2018; Wang et al., 2021), and peanut (Pandey et al., 2017; Clevenger et al., 2018; Luo et al., 2019; Kumar et al., 2020; Luo et al., 2020; Topcu et al., 2021). In peanut, it was used to map genomic loci and candidate genes for the development of diagnostic markers for RRS in 195 RILs obtained by crossing Yuanza 9102 and Xuzhou 68-4 (Luo et al., 2019). A major and stable QTL (*qBWRB02.1*) on chromosome B02 was identified, which was significantly associated with RRS in three environments. Moreover, two SNP sites were confirmed in diverse breeding lines and cultivars. Unlike Yueyou 92, Yuanza 9102 was derived from the wild species *Arachis diogoi* that was resistant to BWD (Janila et al., 2016; Luo et al., 2019). A stable QTL for RRS was finely mapped *via* both linkage mapping and QTL-seq tools in a resistant peanut cultivar (Luo et al., 2020). Two hundred and sixty-eight RILs were sequenced, and the phenotypes of variants from the cross between Xuhua 13 (susceptible) and Zhonghua 6 (resistant) among five environments were evaluated. Using both SSR- and SNP-based genetic maps, the QTL *qBWRB02-1* was identified on chromosome B02 as previously reported (Zhao et al., 2016), and this accounted for 37.79–78.86% phenotypic variation across the five environments. Two adjacent candidate QTL regions in the *qBWRB02-1* locus were segmented into *qBWRB02-1-1* (2.81-4.24 Mb) and *qBWRB02-1-2* (6.54-8.75 Mb) (Luo et al., 2020). *QBWRB02-1-1* accounted for 49.43–68.86% phenotypic variation explained (PVE), which was higher than that for *qBWRB02-1-2* (3.96–6.48% PVE). Moreover, this was validated by competitive allele-specific PCR (KASP) markers in different RILs and natural populations (Luo et al., 2020).

In this study, we utilized a QTL-seq approach to identify concomitant genomic regions, candidate resistance genes and diagnostic markers in a bacterial wilt-resistant peanut variety, Yueyou92. A 7.2 Mb candidate genomic region was elucidated on chromosome 12 significantly associated with RRS. This study reports successful discovery of followed by candidate resistance genes and validated markers for potential use in marker-assisted selection (MAS) for RRS in peanut breeding programs.

## 2 Materials and methods

### 2.1 Plant material and growth

**Figure 1:**
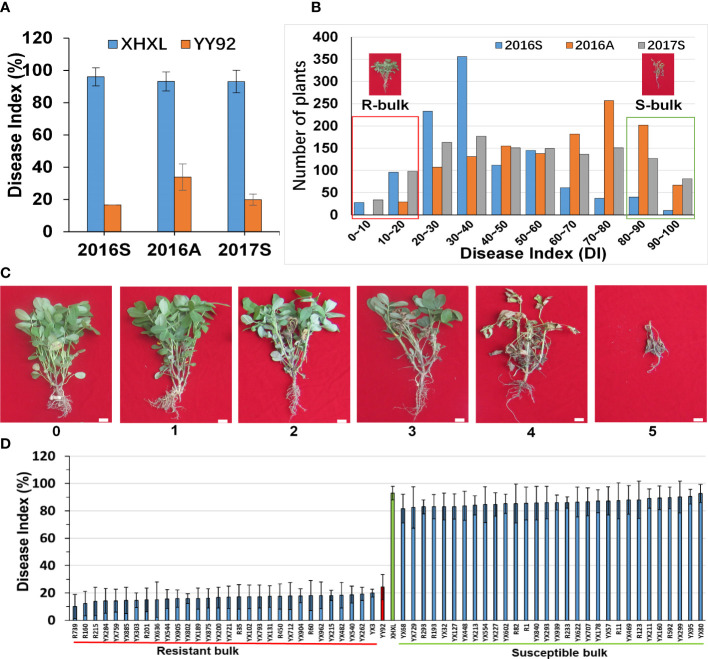
Phenotypic variations and construction of the extreme bulks for resistance to *Ralstonia solanacearum* infections. **(A)** Comparative evaluation of the stability of the resistance between the two parents in the three different crop seasons (2016_Spring, 2016_Autumn, and 2017_Spring). **(B)** Frequency distribution of disease indexes in the RIL population at three different times. The y-axis represented the number of plants, whereas the x-axis represented the disease index. The red dashed box represented the resistant bulk (R-bulk), and the green dashed box represented the susceptible bulk (S-bulk). **(C)** Classification of bacterial wilt disease severity. Disease severity was classified using the following scale: 1 = the inoculated leaflets either had wilt or were absent but the entire plant was intact and lacked wilt; 2 = the main stem/branches of the inoculated leaves had wilt and chlorosis; 3 = the lateral branches of the inoculated leaves had wilt or were faded green, but the main stem was green; 4 = the entire plant had wilted and died, but all its branches were green. 5 = the entire plant had wilted and dried up. Bar: 1 cm. **(D)** Phenotypic variations among the RILs selected for the development of extreme bulks for bacterial wilt resistance. Based on mean values from three environments each with three replications, the 30 RILs with the lowest disease index and the 30 RILs with the highest disease index were used to construct susceptible and resistant bulks.

Yueyou 92 (YY92), a variety that is highly resistant to BWD, was bred by the Guangdong Academy of Agricultural Sciences, China. It stemmed from Xiekangqing, which was resistant to *R. solanacearum* strains from different parts of China. In comparison, Xinhuixiaoli (XHXL) was a Chinese landrace that was highly susceptible to BWD. Their resistance validation was stable during multiple years of field assessment (**Figure 1**). A RIL population containing 581 lines was developed from the cross Yueyou 92 × Xinhuixiaoli using the single seed descent (SSD) method. A total of 581 F_13_ RILs were used for trait mapping for RRS. To assess the diagnostic markers, we utilized 18 resistant and 18 susceptible RILs for genotyping using allele-specific markers. All the RILs and parents were cultivated in a field in Yangzhong County (Sanming, Fujian, China).

### 2.2 Pathogen inoculation and resistance phenotyping

The 581 RILs were evaluated for RRS in three independent crop seasons i.e., in 2016 spring and autumn (2016S and 2016A) and in 2017 spring (2017S). The RILs of F_11_, F_12_, and F_13_ generations and parents were cultivated in two-row plots with 20 seeds in each season. One-month to 40-day-old RILs seedlings were inoculated *via* a previously described artificial method (Zhao et al., 2016; Zhang et al., 2017). Resistance phenotyping of the RILs was performed 25 days after inoculation in the different seasons. Disease symptoms were classified into six disease severity ratings (**Figure 1C**): (0) = the inoculated leaflets either remained green or were yellow at inoculating sites, but the entire plant was intact and lacked wilt; (1) = the inoculated leaflets had either wilted or fallen off, but the entire plant was intact and lacked wilt; (2) = the main stem/branches of the inoculated leaves had wilt and chlorosis; (3) = the leaves of non-inoculated branches had wilt or were faded green, but the main stem was green; (4) = the entire plant had wilted and died, and all its branches were greenish; and (5) = the entire plant had wilted, dried, and was brownish. The disease index (DI) was calculated using the following formula:


$$\text{Disease index}=\frac{\sum_{0}^{5}x_{i}y_{i}}{x_{max}\sum y_{i}}\times 100\%$$

Where,*x*_*i*_ : disease grade value, *x*_*max*_ : the highest disease grade value, and *y*_*i*_: the number of diseased plants corresponding to the disease rating.

The average DI was calculated for the three replications in a single environment. Statistical analysis of variance (ANOVA) was performed using the DPS7.5 software (Date Processing System, Science Press, China), Values are expressed as the mean ± standard deviation or standard error as indicated. Differences between groups were evaluated using one-way ANOVA. Statistical significance was set at P<0.05.

### 2.3 Extreme bulks construction and whole genome resequencing

The average DI for each RIL was calculated based on phenotyping data from the 2016S, 2016A, and 2017S seasons. We selected 30 resistant and 30 susceptible lines to construct the extreme R/S pool. To develop the resistant bulk (R-Bulk) for RRS, we selected 30 RILs with a low mean disease index and pooled the same amount of DNA from each into one. Similarly, DNA samples of 30 RILs with a high mean disease index were pooled to construct the susceptible bulk (S-Bulk) for RRS. The genomic DNA of these two extreme pools and those of the two parents was used to construct DNA sequencing libraries. Paired-end reads (151 bp) of four libraries were generated *via* the Illumina HiSeq 2500 platform (Illumina, Inc., USA) with a sequencing depth of approximately 30× of the cultivated peanut genome (~2.7 Gb) for each pool and about 40× for parental plants. The raw sequencing data of the four libraries have been deposited in the NCBI Sequence Read Archive (SRA) under the BioProject ID PRJNA851221.

### 2.4 SNP/InDel genotype detection and annotation

A QTL-seq approach was used to identify the QTLs for RRS (**Figure S1**) (Takagi et al., 2013). The quality of re-sequenced raw reads from the four libraries was checked. Low-quality reads (those with a proportion of uncalled bases >5%) and adapter sequences were culled. High-quality reads (those with more than 95% nucleotide base calls and high Phred quality scores) were aligned and mapped to the reference genome using the BWA software package (http://bio-bwa.sourceforge.net/) with the default parameters (Li and Durbin, 2009). For further analysis, the genome sequences of allotetraploid progenitors of the cultivated peanut *Arachis hypogaea* (Shitouqi) were downloaded from the Peanut Genome Resource website (http://peanutgr.fafu.edu.cn/) and used as the reference sequences. Duplicated reads were identified and filtered using Picard (http://broadinstitute.github.io/picard/) after mapping the clean reads to the reference genome. To determine the locations and effects of the SNP/InDel variants, we detected and filtered variants in the four libraries using the Genome Analysis Toolkit (GATK, https://software.broadinstitute.org/gatk/) (Cingolani et al., 2012) and annotated using SnpEff software (V.5.0e; https://pcingola.github.io/SnpEff/) (Reumers et al., 2012).

### 2.5 Identification of candidate genomic regions

To identify the candidate genomic regions associated with RRS, we further filtered high-quality reads from S-bulk and R-bulk libraries by removing unpaired reads. To equalize the number of reads from each bulk, the filtered reads of the R-bulk were randomly reduced to the same number of the filtered reads of the S-bulk. During the analysis, the SNP sites with genotypes that differed between the two bulks were used to calculate both the sequencing depth of each base in the different bulks and the Euclidean Distance (ED) value of each site. To eliminate the background noise, the original ED value was processed by power. In this study, the fifth power of the original ED was taken as the correlation value to eliminate the background noise. Then the distance method was used to fit the ED value. For every SNP and InDel in each bulk, ED values were calculated with the formula:


$$ED=\sqrt{{\left(A_{R-bulk}-A_{S-bulk}\right)}^{2}+{\left(C_{R-bulk}-C_{S-bulk}\right)}^{2}+{\left(G_{R-bulk}-G_{S-bulk}\right)}^{2}+{\left(T_{R-bulk}-T_{S-bulk}\right)}^{2}}$$

Each A, G, C, and T letter represented the frequency of its corresponding DNA nucleotide in the resistant and susceptible bulks. The higher the ED value, the stronger the association between the variant with the target characteristic.

The SNP index value was calculated as follows:


$$\text{SNP}-\text{index}(\text{aa})=\frac{Maa}{Maa+Paa}$$

$$\text{SNP}-\text{index}(\text{ab})=\frac{Mab}{Mab+Pab}$$

ΔSNP-index = SNP-index (aa)- SNP-index (ab)

ΔSNP-index was calculated by subtracting the SNP-index of the R-bulk from the SNP-index of the S-bulk. SNP-index plots were generated using sliding window analysis with a window size of 2 Mb and increments of 50 Kb. The SNPs with SNP-index <0.3 or read depth<10 in both bulks were culled (**Supplementary Figure 1**). The SNP index of remaining SNPs as calculated from each bulk was physically plotted onto the 20 cultivated peanut chromosomes. ΔSNP index was calculated by subtracting the SNP index of the resistant bulk from the SNP index of the susceptible bulk. Notably, only those SNPs that had homozygous alleles in both bulks were selected for ΔSNP index calculation. Furthermore, SNP positions were considered as the causal SNPs responsible for the trait of interest if they passed the criterion ΔSNP index = -1. ΔSNP index = -1 indicated that the allele called in resistant bulk was the same as that of the resistant parent while an alternate base was called in susceptible bulk (**Supplementary Figure 3**). This analysis was also used in the InDel correlation analysis (**Supplementary Figure 4**). The DISTANCE method was used to fit ΔSNP-indexes and ED values, and the regions above the correlation threshold value (add value) were selected as those related to traits.

The candidate genomic regions related to RRS had the following significant ΔSNP/InDel index requirements: ΔSNP/InDel index significantly deviated from the statistical confidence intervals under the null hypothesis of no QTLs at a *P* < 0.01 level, and SNP/InDel-index significantly deviated from 0.5 in both bulks. Moreover, the ED values for SNP and InDel were remarkably higher than 0.29 and 0.28, respectively. Finally, the two sets of genomic regions identified from the S-bulk and R-bulk assemblies were combined and considered the genomic regions associated with RRS.

### 2.6 Diagnostic marker development and validation

To validate the identified genomic regions for RRS, SNPs with different alleles in both bulks and near the intersection terminal were identified and a special marker was developed to narrow the candidate region. For the RIL lines under *R. solanacearum* treatments and with the highest and lowest DI values, we randomly selected 18 of each of these two groups, then extracted DNA from them as well as the parents and the other selected samples. The total volume for the PCR reaction was 20 μl, comprising DNA template: 50 ng, 2×PCR Master Mix: 5 μl, forward primer: 10 μM, and reverse primer: 10 μM. The PCR cycling conditions were as follows: 94°C, 3 min; 30–35 cycles of 94°C, 30 s; 56°C, 30 s; 72°C, 30 s; final extension at 72°C, 10 min. After PCR amplification, the targeted amplicons were identified *via* 1.2% agarose gel electrophoresis.

## 3 Results

### 3.1 Phenotype diversity and construction of extreme RRS bulks

To investigate variation in RRS levels of cultivated peanuts, we utilized the resistant “Yueyou 92” (RP) and susceptible “Xinhuixiaoli” (SP) varieties as parents to create multiple generations of segregating RILs populations (**Figure 1A**). The resistance rate was evaluated based on the severity of *R. solanacearum* infections in RILs, which was calculated as a disease index (DI). The DI value of Yueyou 92 was significantly lower than that of Xinhuixiaoli in three consecutive crop seasons (**Figure 1A** and **Supplementary Table 1**). The RILs population had a wide segregation of phenotype variations that formed two peaks of resistance distributions, displaying the main QTLs for RRS regulation (**Figure 1B** and **Supplementary Table 1**). Based on the mean values of the disease index in the three field environments, the 30 RILs with the lowest disease index (10.22–20.00%) and the 30 RILs with the highest index (81.68–92.79%) were selected for construction of the resistant (R-bulk) and susceptible bulks (S-bulk) respectively (**Figure 1D** and **Supplementary Table 1**). Furthermore, phenotypic identification of R- and S-bulk resistance in the greenhouse was like that in the field environment (**Supplementary Figure 1**).

### 3.2 Genome sequencing and SNP/InDel discovery and evaluation

Whole genome sequencing of the parents and the bulks DNA samples was performed on the Illumina HiSeq platform. A total of 114.67 and 103.10 Gb reads were generated for Yueyou 92 and Xinhuixiaoli, and 108.38 and 96.92 Gb for R-bulk and S-bulk respectively. Approximately 97.69% of the reads correctly mapped to the cultivated peanut cv. Shitouqi reference genome (**Table 1**). An average coverage depth of 42× and 37× of the reference genome was achieved by Yueyou 92 and Xinhuixiaoli reads respectively, and 38× and 34× depth for the R-bulk and S-bulk, respectively (**Table 1**). The mapping results showed that the genome was evenly covered, indicating that the sequencing randomness was good (**Supplementary Figure 2**).

**Table 1:** Summary of whole genome re-sequencing of parents and bulk lines for bacterial wilt resistance.

Sample ID	Genotype/bulks	Total_reads	Clean reads	Clean_Base	Q30(%)	GC(%)	Average depth(X)	Genome coverage ration_1X (%)	Genome coverage ration_5X (%)	Genome coverage ration_10X (%)	Mapped(%)	Properly_mapped(%)
RP	Resistant parent	467,742,004	382,713,001	114,670,878,822	89.89%	36.08%	42	97.72%	97.01%	96.42%	99.55%	96.59%
SP	Susceptible parent	602,985,958	344,100,662	103,101,745,626	90.68%	35.86%	37	97.42%	96.62%	95.77%	99.72%	96.31%
R-bulk	Resistant bulk	602,985,958	361,696,407	108,373,962,086	90.94%	35.99%	38	97.83%	97.11%	96.34%	99.74%	96.23%
S-bulk	Susceptible bulk	630,443,472	323,474,233	96,921,445,922	91.27%	35.94%	34	97.77%	96.99%	95.98%	99.74%	96.84%

The short reads of parents and the extreme bulks were aligned to the genome sequences of cultivated peanut, cv. Shitouqi, Arachis hypogaea Linn (Peanut Genome Resource: http://peanutgr.fafu.edu.cn/).

SNPs/InDels were detected and extracted by the GATK software package. A total of 585,258 SNPs and 167,249 InDels were detected between two parents and 126,900 SNPs and 46,013 InDels were detected between the extreme pools, respectively. The occurrence of the SNPs was 3.5 times more than that of the InDels (**Supplementary Table 2**; **Supplementary Figure 3**). After filtering, 381,642 and 98,918 high-quality and homozygous SNP and InDel sites were respectively obtained (**Supplementary Table S3**). Based on the annotations, 72.7% and 55.4% of the SNPs and InDels, respectively, were in the intergenic region between the extreme pools. Approximately 15% of SNPs and 25% of InDels were upstream and downstream of genes, ~10% of variants in introns, and only ~3% of SNPs and 2.6% of InDels were in the coding region of the two bulks. About 34.6% and 54.7% of the SNPs in the CDS region caused synonymous and nonsynonymous coding variants, respectively. Similarly, Approximately 22.0% of the InDels in the CDS region caused frameshift variants (**Supplementary Tables 2**, **3**).

### 3.3 Candidate genomic regions for RRS

**Figure 2:**
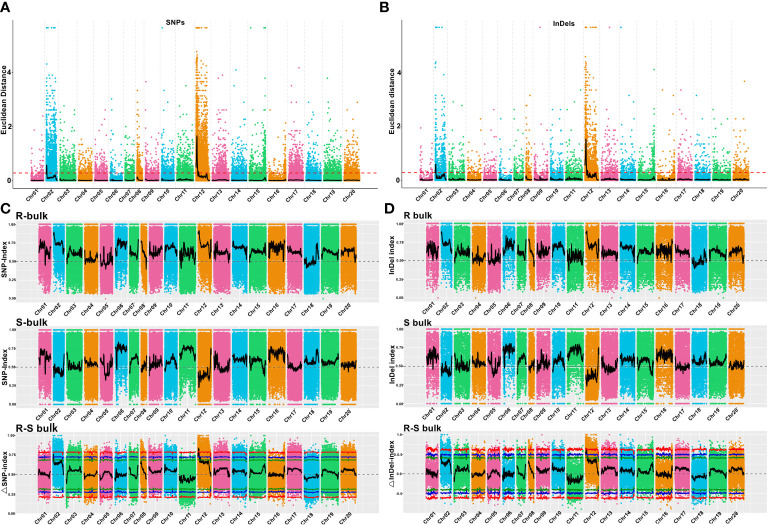
Euclidean distance (ED) value distribution of SNPs/InDels and SNP/InDel-index of R- and S-bulks and Δ(SNP/InDel-index) plots generated by sliding-window analyses of 20 cultivated peanut chromosomes. **(A, B)** Euclidean distance (ED**)** value distribution of SNPs and InDels in 20 chromosomes. The x-axis represents the chromosome name, the colored dots represent ED values, the black lines represent the fitted ED values (with 2Mb windows sliding in 10 kb steps), and the red dotted lines represent associated thresholds. **(C, D)** SNP/InDel-index of R- and S-bulks and Δ(SNP/InDel-index) plots generated by sliding-window analysis of cultivated peanut chromosomes. The physical positions of chromosomes are displayed on the X-axis and the average SNP/InDel-index in each 2-Mb physical interval with a 10-kb sliding window is displayed on the Y-axis. Two candidate genomic regions (marked in yellow) were defined using the criteria: average SNP/InDel-index > 0.9 in the R-bulks and average *P* < 0.05. The red, blue, and green lines represent the thresholds at confidence levels of 0.99, 0.95, and 0.90, respectively.

**Figure 3:**
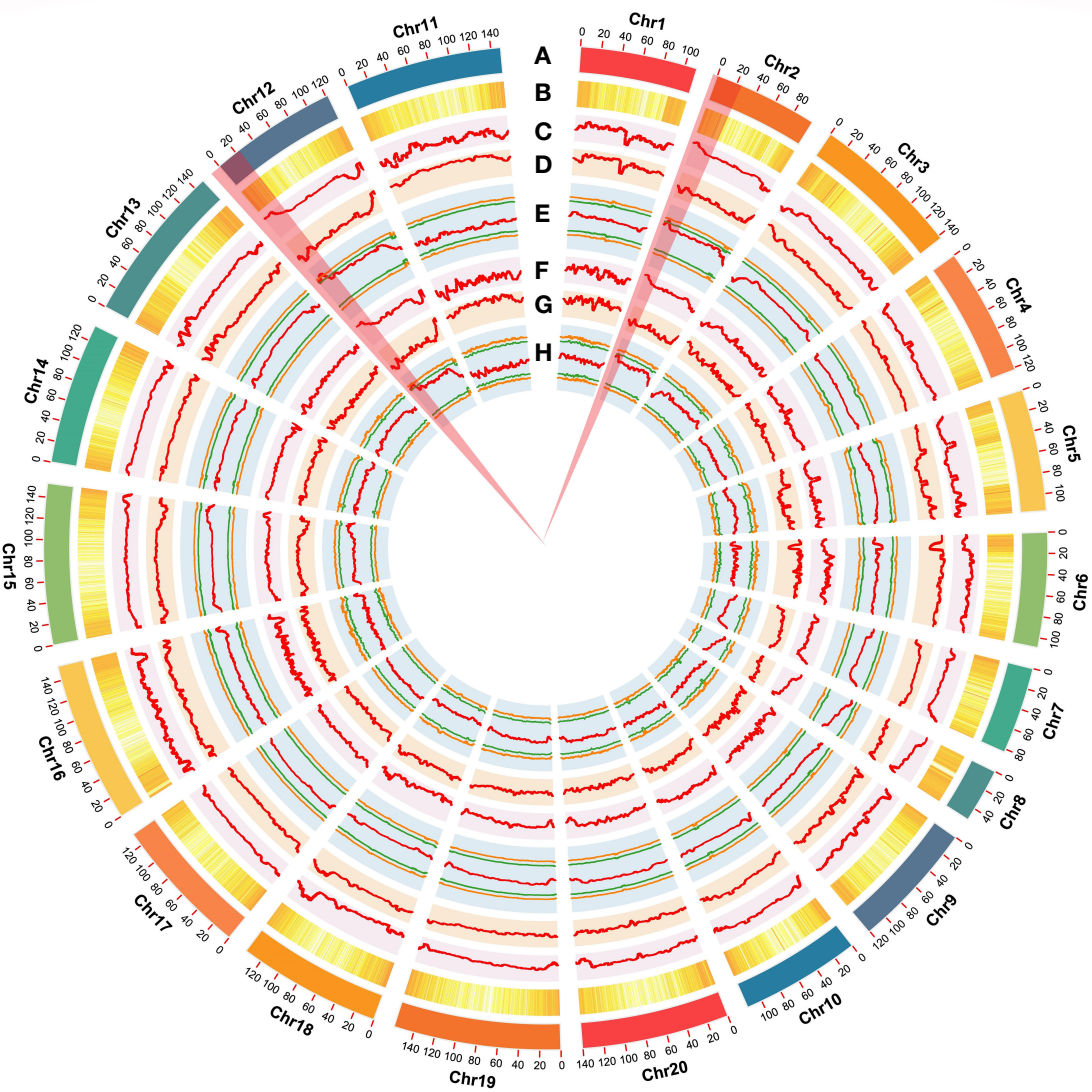
Genome-wide summary of the putative genomic regions associated with resistance to *Ralstonia solanacearum* infections. **(A)** Chromosomes of the cultivated peanut reference genome. **(B)** The genome-wide density of total genes. **(C, D)** Plots of SNP-index of R- and S-bulks generated by sliding-window analyses of cultivated peanut chromosomes. **(E)** ΔSNP-index plot generated by using the Shitouqi assembly as a reference genome. Label definitions from outside to inside: upper probability values at 99% (orange) and 95% (green) confidence levels. ΔSNP-index, lower probability values at 95% (green), and 99% (orange) confidence levels. **(F, G)** InDel-index of R- and S-bulks plots generated by sliding-window analyses of cultivated peanut chromosomes. **(H)** ΔInDel-index plot generated by using the Shitouqi assembly as a reference genome. Label definitions from outside to inside: upper probability values at 99% (orange) and 95% (green) confidence levels. ΔSNP-index, lower probability values at 95% (green) and 99% (orange) confidence levels.

Using the genome sequences of *Arachis hypogaea* (Shitouqi) as reference, the Euclidean distance (ED) and SNP index, including the ΔSNP-index, were calculated for each genome-wide high-quality SNPs, from RP and SP (**Figure 2**; **Supplementary Figure 6**; **Supplementary Figure 8**). Then, candidate genomic regions for RRS were identified based on ED and ΔSNP-index plots through sliding window analysis of deviations from the threshold value at a 99% confidence level. By using an ED association algorithm, a major peak on Chr12 was identified for RRS, spanning 0–15.19 Mb with an ED > 0.29 (*P*<0.01) for the SNP. A 6.40 Mb (0.77–7.17 Mb) interval on Chr02 was also identified. By SNP-index and ΔSNP-index, only a genomic interval of 5.83 Mb (4.16–9.99 Mb) on Chr12 deviated from the threshold with the confidence level of *P*<0.01 (**Figure 2**; **Supplementary Figure 7**; **Supplementary Figure 9**), indicating the interval on Chr12 as the main region controlling the RRS. Moreover, the ED and InDel-indexes (referring to principles of ΔSNP-index) for each identified genomic InDel were calculated for RP and SP. The regions of similarity were confirmed at intervals of 0-7.0 Mb and 0-15 Mb on Chr02 and Chr12, respectively, for ED mapping and 7.49–9.99 Mb on Chr12 for InDel-index association (**Supplementary Figures 6**-**9**). Once more, the candidate region on Chr12 was robust with a *P*<0.01 confidence level for both methods. As SNP-indexes enabled fine mapping, the 7.2 Mb (1.8–9.0 Mb) and 5.83 Mb (4.16–9.99 Mb) intervals on Chr12 were collectively identified as candidate region associated with RRS, at 95% and 99% confidence levels, respectively (**Figure 3**).

### 3.4 Genetic confirmation of candidate genomic region

To confirm the candidate genomic regions associated with RRS by the QTL-seq approach, we remapped the linkage group (LG) of the existing genetic map to the previously published and newly developed SNP markers. The QTL map had two QTLs located in LG1 (ChrB02) and LG10 in F_2_, which explained 21% and 12% of phenotype variations, and one QTL with two adjoining peaks in LG1 (ChrB02) in F_8_ (Zhao et al., 2016). The QTL on ChrB02 was located between SNP markers SNP79 and SNP129 in LG1, for which the LOD value was 3.91 and over 6.22, respectively (Zhao et al., 2016). We remapped the SNP markers to the cultivated peanut reference genome. The SNP79 and SNP129 markers were at 1.2 Mb and 9.2 Mb on Chr12, respectively, corroborating identified candidate genomic region through QTL-seq approach (**Supplementary Figure 10**). Recently, QTL analyses based on SLAF-seq were conducted to detect the candidate QTL region that confers RRS, and the concomitant genotyping and phenotyping data was used for mapping. This resulted in the identification of a consistent region between the SNP marker loci Marker7969064 and Marker7795914 (unpublished data), which explained 45% of the phenotype variations. Marker7969064 and Marke7795914 were located at 6.2 Mb and 8.7 Mb on Chr12, respectively (**Supplementary Figure 10**). The candidate genomic region associated with RRS as per QTL mapping corroborated that from the QTL-seq method. These results supported QTL-seq results, which revealed the candidate genomic region is associated with RRS.

### 3.5 Candidate genes associated with RRS

**Figure 4:**
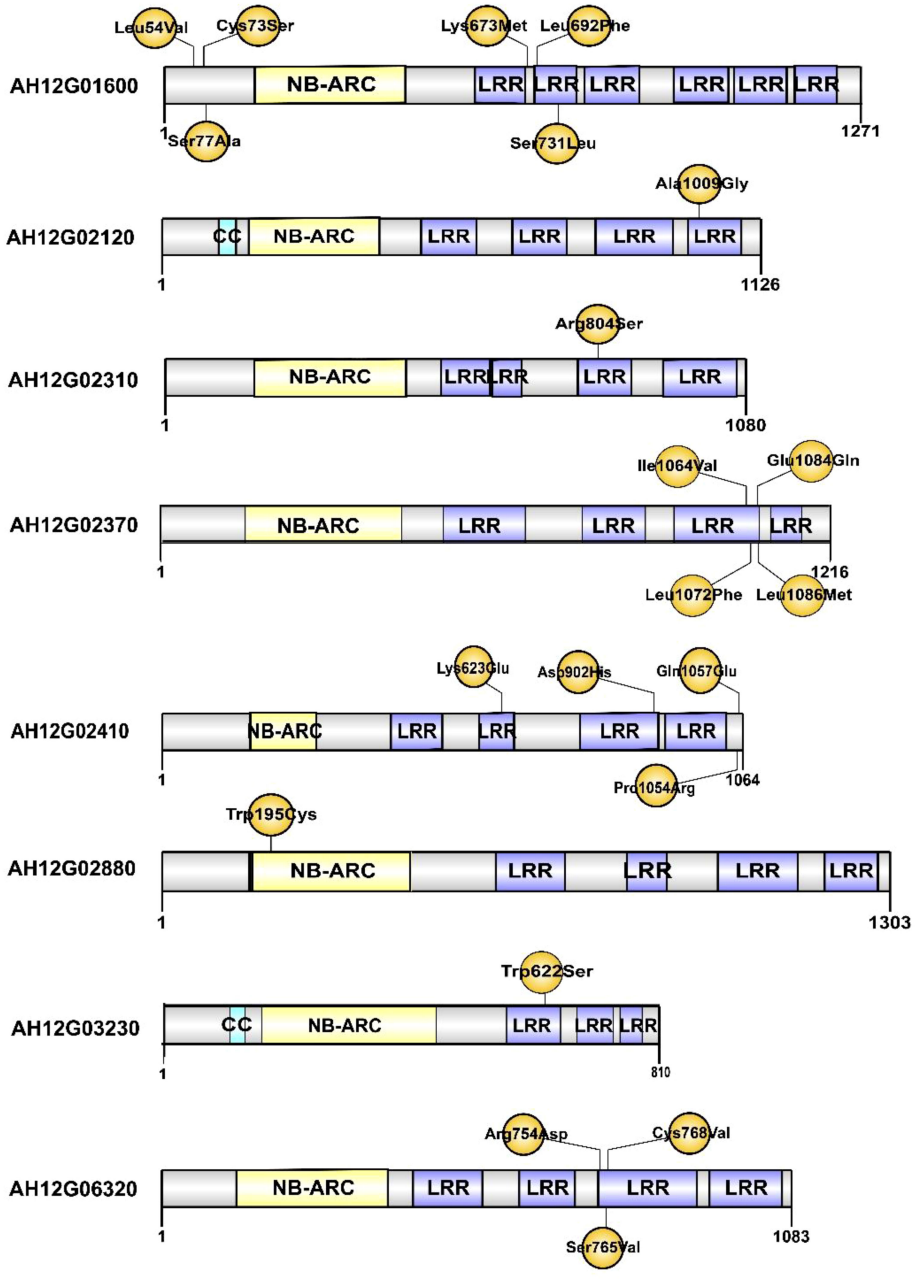
The putative resistance-related proteins associated with resistance to *Ralstonia solanacearum* infections. CC: Coil coiled. NB-ARC: Nucleotide-binding adapter shared by APAF-1 R proteins and CED-4. LRR: Leucine-rich repeat domain. The positions of amino acid changes caused by nonsynonymous SNPs are shown in yellow.

To narrow down the genomic regions and validate effective SNPs associated with RRS on Chr12, we selected a genomic region spanning 7.2 Mb on Chr12 and with 1807 effective SNPs and 629 InDels. Function annotation analysis of the 1807 SNPs revealed 503 intergenic SNPs; 461 intronic; 357 and 225 that were upstream and downstream of genes, respectively; two, six, and eight in 5’ UTR, 3’ UTR, and splice site regions, respectively; and 67 synonymous and 180 nonsynonymous (two resulted in stop codons) (**Supplementary Table 4**). Notably, 22 genes with nonsynonymous SNPs were predicted to encode for the NBS-LRR type disease resistance proteins, including *AH12G01510, AH12G01540, AH12G01550, AH12G01560, AH12G01570, AH12G01600, AH12G01900, AH12G01920, AH12G01980, AH12G02020, AH12G02090, AH12G02120, AH12G02130, AH12G02310, AH12G02330, AH12G02370, AH12G02390, AH12G02410. AH12G02880, AH12G03230, AH12G03600* and *AH12G06320* (**Table 2**). The *AH12G01460* and *AH12G06300* encode a receptor-like kinase protein and exocyst subunit *Exo70* family protein B2 subunit respectively. *AH12G03290* and *AH12G05320* both encode Serine/threonine-protein phosphatase 7. The other putative candidate genes encoded various kinds of proteins (**Table 3**). Notably, eight of 22 candidate NBS-LRR resistant genes were identified with high confidence as important candidate genes with the ΔSNP values above 0.60 (**Figure 4**). Moreover, among the 629 InDels, 152 and 189 were in the intergenic and intronic regions, respectively, 146 and 114 were upstream and downstream of genes, respectively, five and four in 5’ UTR and 3’ UTR regions, respectively, nine and five resulted in frame shifts and codon changes respectively (**Supplementary Table 5**). The 180 nonsynonymous SNPs affected 75 putative candidate genes associated with RRS (**Table 2**), whereas 14 InDels affected 11 genes (**Table 3**). Among them, six in NBS-LRR genes *AH12G01920*, *AH12G01980*, *AH12G02090*, *AH12G02390*, *AH12G02440*, *AH12G02600* affected the encoded functions as well as ΔSNP-index results (**Table 3**). Taken together, these results support the hypothesis that the six NBS-LRR resistance genes might act as the candidate genes related to RRS.

**Table 2:** Identification of SNPs in putative candidate genes in the genomic region for resistance to on chromosome 12.

Gene ID	Chromosome	Physical position (bp)	Reference Genome	Alternative site	Resistant bulk (RB) base	Number of reads covering the site (X coverage) in resistant bulk (RB)	RB Depths of Ref, Alt	SNP-index of RB	Susceptible bulk (SB) base	Number of reads covering the site (X coverage) in susceptible bulk(SB)	SB Depths of Ref, Alt	SNP-index of SB	Delta SNP-index (RB SNP-index-SB SNP-index)	SNP substitution effect	Amino acid change	U95 (95% confidence interval upper side)	L95 (95% confidence interval lower side)	U99 (99% confidence interval upper side)	L99 (99% confidence interval lower side)	Gene function
AH12G01450	Chr12	1817271	A	G	R	25	21,4	0.84	R	10	1,9	0.10	0.74	NON_SYNONYMOUS_CODING	Tct/Cct	0.459825	-0.460953	0.585964	-0.588628	AT-rich interactive domain-containing protein 1
AH12G01450	Chr12	1823428	C	T	Y	29	25,4	0.86	Y	15	3,12	0.20	0.66	NON_SYNONYMOUS_CODING	Gaa/Aaa	0.459812	-0.460941	0.585948	-0.588612	AT-rich interactive domain-containing protein 1
AH12G01460	Chr12	1832855	C	T	Y	19	15,4	0.79	Y	10	1,9	0.10	0.69	NON_SYNONYMOUS_CODING	Gaa/Aaa	0.4598	-0.460929	0.585932	-0.588596	Receptor-like protein B6:U6kinase 4
AH12G01510	Chr12	1864236	C	G	C	19	19,0	1.00	S	8	6,2	0.75	0.25	NON_SYNONYMOUS_CODING	Caa/Gaa	0.459761	-0.460891	0.585885	-0.588548	Putative disease resistance RPP13-like protein 1
AH12G01540	Chr12	1888227	G	C	G	17	16,1	0.94	S	5	3,2	0.60	0.34	NON_SYNONYMOUS_CODING	Gga/Cga	0.459735	-0.460866	0.585852	-0.588516	Putative disease resistance RPP13-like protein 1
AH12G01550	Chr12	1889601	G	A	G	19	19,0	1.00	R	5	2,3	0.40	0.60	NON_SYNONYMOUS_CODING	Gaa/Aaa	0.459735	-0.460866	0.585852	-0.588516	Putative disease resistance RPP13-like protein 1
AH12G01550	Chr12	1889607	A	C	A	20	20,0	1.00	M	6	2,4	0.33	0.67	NON_SYNONYMOUS_CODING	Aat/Cat	0.459735	-0.460866	0.585852	-0.588516	Putative disease resistance RPP13-like protein 1
AH12G01550	Chr12	1889737	T	A	T	22	22,0	1.00	W	5	3,2	0.60	0.40	NON_SYNONYMOUS_CODING	gTt/gAt	0.459735	-0.460866	0.585852	-0.588516	Putative disease resistance RPP13-like protein 1
AH12G01560	Chr12	1889759	T	A	T	17	17,0	1.00	W	5	4,1	0.80	0.20	NON_SYNONYMOUS_CODING	gaT/gaA	0.459735	-0.460866	0.585852	-0.588516	Putative disease resistance RPP13-like protein 1
AH12G01570	Chr12	1896280	A	G	R	21	19,2	0.90	R	5	2,3	0.40	0.50	NON_SYNONYMOUS_CODING	gAt/gGt	0.459722	-0.460854	0.585837	-0.5885	Putative disease resistance RPP13-like protein 1
AH12G01570	Chr12	1896290	G	A	R	22	20,2	0.91	R	5	2,3	0.40	0.51	NON_SYNONYMOUS_CODING	atG/atA	0.459722	-0.460854	0.585837	-0.5885	Putative disease resistance RPP13-like protein 1
AH12G01570	Chr12	1896327	G	T	G	17	16,1	0.94	K	4	1,3	0.25	0.69	NON_SYNONYMOUS_CODING	Ggc/Tgc	0.459722	-0.460854	0.585837	-0.5885	Putative disease resistance RPP13-like protein 1
AH12G01570	Chr12	1896685	G	A	G	14	13,1	0.93	R	5	3,2	0.60	0.33	NON_SYNONYMOUS_CODING	cGa/cAa	0.459722	-0.460854	0.585837	-0.5885	Putative disease resistance RPP13-like protein 1
AH12G01570	Chr12	1896707	C	A	C	16	15,1	0.94	M	5	3,2	0.60	0.34	NON_SYNONYMOUS_CODING	ttC/ttA	0.459722	-0.460854	0.585837	-0.5885	Putative disease resistance RPP13-like protein 1
AH12G01600	Chr12	1929770	C	G	C	23	23,0	1.00	S	11	3,8	0.27	0.73	NON_SYNONYMOUS_CODING	Ctt/Gtt	0.459683	-0.460815	0.585788	-0.588452	Putative disease resistance RPP13-like protein 1
AH12G01600	Chr12	1929827	T	A	T	22	22,0	1.00	W	6	2,4	0.33	0.67	NON_SYNONYMOUS_CODING	Tgt/Agt	0.459683	-0.460815	0.585788	-0.588452	Putative disease resistance RPP13-like protein 1
AH12G01600	Chr12	1929839	T	G	T	20	20,0	1.00	K	6	2,4	0.33	0.67	NON_SYNONYMOUS_CODING	Tct/Gct	0.459683	-0.460815	0.585788	-0.588452	Putative disease resistance RPP13-like protein 1
AH12G01600	Chr12	1931628	A	T	A	15	15,0	1.00	W	5	2,3	0.40	0.60	NON_SYNONYMOUS_CODING	aAg/aTg	0.45967	-0.460802	0.585771	-0.588435	Putative disease resistance RPP13-like protein 1
AH12G01600	Chr12	1931686	A	C	A	12	12,0	1.00	M	11	3,8	0.27	0.73	NON_SYNONYMOUS_CODING	ttA/ttC	0.45967	-0.460802	0.585771	-0.588435	Putative disease resistance RPP13-like protein 1
AH12G01600	Chr12	1931802	C	T	C	13	13,0	1.00	Y	9	3,6	0.33	0.67	NON_SYNONYMOUS_CODING	tCg/tTg	0.45967	-0.460802	0.585771	-0.588435	Putative disease resistance RPP13-like protein 1
AH12G01630	Chr12	1962973	T	C	Y	19	14,5	0.74	Y	14	4,10	0.29	0.45	NON_SYNONYMOUS_CODING	aAa/aGa	0.45963	-0.460764	0.585722	-0.588387	Pentatricopeptide repeat-containing protein
AH12G01670	Chr12	2019013	A	C	A	24	23,1	0.96	M	21	14,7	0.67	0.29	NON_SYNONYMOUS_CODING	aaA/aaC	0.459384	-0.460528	0.58544	-0.588095	HXXXD-type acyl-transferase family protein
AH12G01670	Chr12	2019053	A	C	A	25	24,1	0.96	M	19	13,6	0.68	0.28	NON_SYNONYMOUS_CODING	Aca/Cca	0.459384	-0.460528	0.58544	-0.588095	HXXXD-type acyl-transferase family protein
AH12G01670	Chr12	2019086	T	C	Y	22	20,2	0.91	Y	23	16,7	0.70	0.21	NON_SYNONYMOUS_CODING	Ttt/Ctt	0.459384	-0.460528	0.58544	-0.588095	HXXXD-type acyl-transferase family protein
AH12G01670	Chr12	2019242	A	G	A	16	15,1	0.94	R	20	18,2	0.90	0.04	NON_SYNONYMOUS_CODING	Aat/Gat	0.459384	-0.460528	0.58544	-0.588095	HXXXD-type acyl-transferase family protein
AH12G01670	Chr12	2019615	A	G	A	21	21,0	1.00	R	14	13,1	0.93	0.07	NON_SYNONYMOUS_CODING	aAg/aGg	0.459384	-0.460528	0.58544	-0.588095	HXXXD-type acyl-transferase family protein
AH12G01670	Chr12	2019677	A	G	R	27	25,2	0.93	R	16	11,5	0.69	0.24	NON_SYNONYMOUS_CODING	Aat/Gat	0.459384	-0.460528	0.58544	-0.588095	HXXXD-type acyl-transferase family protein
AH12G01670	Chr12	2019736	C	G	S	24	22,2	0.92	S	20	13,7	0.65	0.27	NON_SYNONYMOUS_CODING	ttC/ttG	0.459384	-0.460528	0.58544	-0.588095	HXXXD-type acyl-transferase family protein
AH12G01670	Chr12	2019773	G	A	G	25	24,1	0.96	R	20	14,6	0.70	0.26	NON_SYNONYMOUS_CODING	Gtt/Att	0.459384	-0.460528	0.58544	-0.588095	HXXXD-type acyl-transferase family protein
AH12G01670	Chr12	2019997	T	G	T	21	21,0	1.00	K	11	6,5	0.55	0.45	NON_SYNONYMOUS_CODING	atT/atG	0.459384	-0.460528	0.58544	-0.588095	HXXXD-type acyl-transferase family protein
AH12G01670	Chr12	2020027	G	T	G	21	21,0	1.00	K	11	9,2	0.82	0.18	NON_SYNONYMOUS_CODING	agG/agT	0.459292	-0.46044	0.585336	-0.587987	HXXXD-type acyl-transferase family protein
AH12G01670	Chr12	2020059	G	T	G	21	21,0	1.00	K	9	7,2	0.78	0.22	NON_SYNONYMOUS_CODING	aGa/aTa	0.459292	-0.46044	0.585336	-0.587987	HXXXD-type acyl-transferase family protein
AH12G01670	Chr12	2020110	T	C	T	22	21,1	0.95	Y	22	15,7	0.68	0.27	NON_SYNONYMOUS_CODING	gTa/gCa	0.459292	-0.46044	0.585336	-0.587987	HXXXD-type acyl-transferase family protein
AH12G01670	Chr12	2020205	G	A	R	27	24,3	0.89	R	31	17,14	0.55	0.34	NON_SYNONYMOUS_CODING	Gta/Ata	0.459292	-0.46044	0.585336	-0.587987	HXXXD-type acyl-transferase family protein
AH12G01670	Chr12	2020297	G	C	S	30	27,3	0.90	S	28	15,13	0.54	0.36	NON_SYNONYMOUS_CODING	gaG/gaC	0.459292	-0.46044	0.585336	-0.587987	HXXXD-type acyl-transferase family protein
AH12G01670	Chr12	2020334	A	G	A	33	30,3	0.91	R	30	19,11	0.63	0.28	NON_SYNONYMOUS_CODING	Act/Gct	0.459292	-0.46044	0.585336	-0.587987	HXXXD-type acyl-transferase family protein
AH12G01670	Chr12	2020367	A	G	A	31	30,1	0.97	R	26	20,6	0.77	0.20	NON_SYNONYMOUS_CODING	Atg/Gtg	0.459292	-0.46044	0.585336	-0.587987	HXXXD-type acyl-transferase family protein
AH12G01670	Chr12	2020380	G	A	G	26	25,1	0.96	R	26	20,6	0.77	0.19	NON_SYNONYMOUS_CODING	aGg/aAg	0.459292	-0.46044	0.585336	-0.587987	HXXXD-type acyl-transferase family protein
AH12G01690	Chr12	2054547	T	C	T	25	24,1	0.96	Y	20	17,3	0.85	0.11	NON_SYNONYMOUS_CODING	Ttt/Ctt	0.459047	-0.460203	0.585059	-0.587699	HXXXD-type acyl-transferase family protein
AH12G01720	Chr12	2106320	T	C	Y	17	13,4	0.76	Y	13	2,11	0.15	0.61	NON_SYNONYMOUS_CODING	Agt/Ggt	0.458788	-0.459954	0.584757	-0.58739	Subtilase family protein
AH12G01780	Chr12	2249782	C	T	C	18	16,2	0.89	Y	17	3,14	0.18	0.71	NON_SYNONYMOUS_CODING	aCa/aTa	0.458144	-0.459339	0.584017	-0.586617	Glycerol-3-phosphate 2-O-acyltransferase 6
AH12G01880	Chr12	2427316	C	T	Y	26	23,3	0.88	Y	13	3,10	0.23	0.65	NON_SYNONYMOUS_CODING	tCc/tTc	0.457359	-0.45859	0.583129	-0.585683	UDP-glycosyltransferase 91A1
AH12G01900	Chr12	2432994	A	G	R	23	20,3	0.87	R	15	2,13	0.13	0.74	NON_SYNONYMOUS_CODING	cAc/cGc	0.457315	-0.458548	0.58308	-0.585627	Putative disease resistance RPP13-like protein 1
AH12G01910	Chr12	2448354	A	G	R	25	22,3	0.88	R	14	1,13	0.07	0.81	NON_SYNONYMOUS_CODING	Aga/Gga	0.457272	-0.458507	0.58303	-0.585572	UDP-glycosyltransferase 91A1
AH12G01920	Chr12	2473114	G	A	R	26	23,3	0.88	R	15	2,13	0.13	0.75	NON_SYNONYMOUS_CODING	gGa/gAa	0.457162	-0.458401	0.582905	-0.585432	Putative disease resistance protein At3g14460
AH12G01920	Chr12	2473124	G	C	S	24	21,3	0.88	S	19	3,16	0.16	0.72	NON_SYNONYMOUS_CODING	tgG/tgC	0.457162	-0.458401	0.582905	-0.585432	Putative disease resistance protein At3g14460
AH12G01980	Chr12	2534393	C	T	Y	19	17,2	0.89	Y	19	17,2	0.89	0.00	NON_SYNONYMOUS_CODING	cCa/cTa	0.456951	-0.458197	0.582663	-0.585158	Putative disease resistance RPP13-like protein 1
AH12G01980	Chr12	2534405	C	T	Y	22	20,2	0.91	Y	18	16,2	0.89	0.02	NON_SYNONYMOUS_CODING	cCt/cTt	0.456951	-0.458197	0.582663	-0.585158	Putative disease resistance RPP13-like protein 1
AH12G01980	Chr12	2534443	A	G	R	16	15,1	0.94	R	17	15,2	0.88	0.06	NON_SYNONYMOUS_CODING	Aga/Gga	0.456951	-0.458197	0.582663	-0.585158	Putative disease resistance RPP13-like protein 1
AH12G01980	Chr12	2534503	C	T	Y	18	16,2	0.89	Y	14	10,4	0.71	0.17	NON_SYNONYMOUS_CODING	Ccc/Tcc	0.456951	-0.458197	0.582663	-0.585158	Putative disease resistance RPP13-like protein 1
AH12G01980	Chr12	2534516	C	T	Y	19	16,3	0.84	Y	13	8,5	0.62	0.23	NON_SYNONYMOUS_CODING	tCg/tTg	0.456951	-0.458197	0.582663	-0.585158	Putative disease resistance RPP13-like protein 1
AH12G01980	Chr12	2534533	C	T	Y	22	18,4	0.82	Y	14	9,5	0.64	0.18	NON_SYNONYMOUS_CODING	Cca/Tca	0.456951	-0.458197	0.582663	-0.585158	Putative disease resistance RPP13-like protein 1
AH12G01980	Chr12	2534560	G	A	R	22	18,4	0.82	R	15	10,5	0.67	0.15	NON_SYNONYMOUS_CODING	Gta/Ata	0.456951	-0.458197	0.582663	-0.585158	Putative disease resistance RPP13-like protein 1
AH12G01980	Chr12	2539578	T	A	T	24	24,0	1.00	W	23	20,3	0.87	0.13	NON_SYNONYMOUS_CODING	Aca/Tca	0.456951	-0.458197	0.582663	-0.585158	Putative disease resistance RPP13-like protein 1
AH12G01980	Chr12	2539652	G	C	G	20	20,0	1.00	S	20	15,5	0.75	0.25	NON_SYNONYMOUS_CODING	Gtc/Ctc	0.456951	-0.458197	0.582663	-0.585158	Putative disease resistance RPP13-like protein 1
AH12G01980	Chr12	2539689	C	A	C	26	26,0	1.00	M	24	15,9	0.63	0.38	NON_SYNONYMOUS_CODING	Cca/Aca	0.456951	-0.458197	0.582663	-0.585158	Putative disease resistance RPP13-like protein 1
AH12G01980	Chr12	2539742	G	C	G	27	27,0	1.00	S	29	20,9	0.69	0.31	NON_SYNONYMOUS_CODING	tCg/tGg	0.456951	-0.458197	0.582663	-0.585158	Putative disease resistance RPP13-like protein 1
AH12G01980	Chr12	2539781	T	A	T	24	24,0	1.00	W	33	22,11	0.67	0.33	NON_SYNONYMOUS_CODING	aAc/aTc	0.456951	-0.458197	0.582663	-0.585158	Putative disease resistance RPP13-like protein 1
AH12G01980	Chr12	2539858	A	T	A	21	20,1	0.95	W	27	18,9	0.67	0.29	NON_SYNONYMOUS_CODING	gaT/gaA	0.456951	-0.458197	0.582663	-0.585158	Putative disease resistance RPP13-like protein 1
AH12G01980	Chr12	2540304	C	G	C	31	31,0	1.00	S	16	12,4	0.75	0.25	NON_SYNONYMOUS_CODING	Gat/Cat	0.45693	-0.458177	0.582639	-0.58513	Putative disease resistance RPP13-like protein 1
AH12G01980	Chr12	2540329	G	C	G	29	29,0	1.00	S	15	11,4	0.73	0.27	NON_SYNONYMOUS_CODING	aaC/aaG	0.45693	-0.458177	0.582639	-0.58513	Putative disease resistance RPP13-like protein 1
AH12G01980	Chr12	2540382	T	G	T	25	25,0	1.00	K	14	11,3	0.79	0.21	NON_SYNONYMOUS_CODING	Aac/Cac	0.45693	-0.458177	0.582639	-0.58513	Putative disease resistance RPP13-like protein 1
AH12G01980	Chr12	2545381	A	C	A	23	22,1	0.96	M	17	14,3	0.82	0.13	NON_SYNONYMOUS_CODING	Tcc/Gcc	0.45693	-0.458177	0.582639	-0.58513	Putative disease resistance RPP13-like protein 1
AH12G01980	Chr12	2545481	A	G	R	23	20,3	0.87	R	18	12,6	0.67	0.20	NON_SYNONYMOUS_CODING	gAc/gGc	0.45693	-0.458177	0.582639	-0.58513	Putative disease resistance RPP13-like protein 1
AH12G01980	Chr12	2545502	C	G	S	26	23,3	0.88	S	15	12,3	0.80	0.08	NON_SYNONYMOUS_CODING	aCt/aGt	0.45693	-0.458177	0.582639	-0.58513	Putative disease resistance RPP13-like protein 1
AH12G02020	Chr12	2547669	C	T	C	13	13,0	1.00	Y	19	15,4	0.79	0.21	NON_SYNONYMOUS_CODING	gGa/gAa	0.45693	-0.458177	0.582639	-0.58513	Putative disease resistance RPP13-like protein 1
AH12G02090	Chr12	2620498	C	T	C	25	25,0	1.00	Y	9	7,2	0.78	0.22	NON_SYNONYMOUS_CODING	Gac/Aac	0.456824	-0.458079	0.582535	-0.584984	Putative disease resistance RPP13-like protein 1
AH12G02090	Chr12	2620504	A	C	A	25	25,0	1.00	M	9	7,2	0.78	0.22	NON_SYNONYMOUS_CODING	Tac/Gac	0.456824	-0.458079	0.582535	-0.584984	Putative disease resistance RPP13-like protein 1
AH12G02090	Chr12	2621513	C	A	M	22	19,3	0.86	M	13	8,5	0.62	0.25	NON_SYNONYMOUS_CODING	aGg/aTg	0.456824	-0.458079	0.582535	-0.584984	Putative disease resistance RPP13-like protein 1
AH12G02120	Chr12	2641657	G	C	S	13	12,1	0.92	S	19	3,16	0.16	0.77	NON_SYNONYMOUS_CODING	gCc/gGc	0.456806	-0.458062	0.582519	-0.584957	Putative disease resistance RPP13-like protein 1
AH12G02130	Chr12	2651310	C	T	Y	13	6,7	0.54	Y	11	8,3	0.27	0.27	NON_SYNONYMOUS_CODING	gGt/gAt	0.456798	-0.458054	0.582513	-0.584944	Putative disease resistance RPP13-like protein 1
AH12G02130	Chr12	2651350	A	C	M	7	4,3	0.43	M	9	7,2	0.22	0.21	NON_SYNONYMOUS_CODING	Ttg/Gtg	0.456798	-0.458054	0.582513	-0.584944	Putative disease resistance RPP13-like protein 1
AH12G02130	Chr12	2651430	T	A	W	9	4,5	0.56	T	10	9,1	0.10	0.46	NON_SYNONYMOUS_CODING	aAt/aTt	0.456798	-0.458054	0.582513	-0.584944	Putative disease resistance RPP13-like protein 1
AH12G02310	Chr12	2777261	G	T	K	11	9,2	0.82	T	14	0,14	0.00	0.82	NON_SYNONYMOUS_CODING	agG/agT	0.456652	-0.457914	0.582398	-0.584766	Putative disease resistance RPP13-like protein 1
AH12G02310	Chr12	2816492	T	G	K	17	13,4	0.76	K	15	2,13	0.13	0.63	STOP_LOST	tAa/tCa	0.456653	-0.457913	0.582412	-0.582412	Putative disease resistance RPP13-like protein 1
AH12G02330	Chr12	2820701	G	A	G	23	23,0	1.00	R	12	11,1	0.92	0.08	NON_SYNONYMOUS_CODING	aGg/aAg	0.456665	-0.457925	0.582427	-0.584779	Putative disease resistance RPP13-like protein 1
AH12G02330	Chr12	2820718	A	C	A	24	24,0	1.00	M	13	11,2	0.85	0.15	NON_SYNONYMOUS_CODING	Atc/Ctc	0.456665	-0.457925	0.582427	-0.584779	Putative disease resistance RPP13-like protein 1
AH12G02330	Chr12	2820776	A	G	A	28	28,0	1.00	R	15	12,3	0.80	0.20	NON_SYNONYMOUS_CODING	gAa/gGa	0.456665	-0.457925	0.582427	-0.584779	Putative disease resistance RPP13-like protein 1
AH12G02330	Chr12	2820802	A	C	A	27	27,0	1.00	M	16	12,4	0.75	0.25	NON_SYNONYMOUS_CODING	Att/Ctt	0.456665	-0.457925	0.582427	-0.584779	Putative disease resistance RPP13-like protein 1
AH12G02330	Chr12	2820833	G	C	G	28	28,0	1.00	S	15	11,4	0.73	0.27	NON_SYNONYMOUS_CODING	gGt/gCt	0.456665	-0.457925	0.582427	-0.584779	Putative disease resistance RPP13-like protein 1
AH12G02330	Chr12	2820846	T	A	T	26	26,0	1.00	W	14	11,3	0.79	0.21	NON_SYNONYMOUS_CODING	agT/agA	0.456665	-0.457925	0.582427	-0.584779	Putative disease resistance RPP13-like protein 1
AH12G02330	Chr12	2820944	G	A	G	22	22,0	1.00	R	11	5,6	0.45	0.55	NON_SYNONYMOUS_CODING	cGg/cAg	0.456665	-0.457925	0.582427	-0.584779	Putative disease resistance RPP13-like protein 1
AH12G02330	Chr12	2822450	A	C	M	23	20,3	0.87	M	23	4,19	0.17	0.70	NON_SYNONYMOUS_CODING	gAt/gCt	0.456665	-0.457925	0.582427	-0.584779	Putative disease resistance RPP13-like protein 1
AH12G02330	Chr12	2822465	A	G	R	21	18,3	0.86	R	22	5,17	0.23	0.63	NON_SYNONYMOUS_CODING	cAt/cGt	0.456665	-0.457925	0.582427	-0.584779	Putative disease resistance RPP13-like protein 1
AH12G02370	Chr12	2823195	T	C	T	19	19,0	1.00	Y	6	2,4	0.33	0.67	NON_SYNONYMOUS_CODING	tAt/tGt	0.456665	-0.457925	0.582427	-0.584779	Putative disease resistance RPP13-like protein 1
AH12G02370	Chr12	2823222	G	A	G	20	20,0	1.00	R	7	2,5	0.29	0.71	NON_SYNONYMOUS_CODING	Gca/Cca	0.456665	-0.457925	0.582427	-0.584779	Putative disease resistance RPP13-like protein 1
AH12G02370	Chr12	2823262	G	C	G	26	26,0	1.00	S	5	1,4	0.20	0.80	NON_SYNONYMOUS_CODING	Gaa/Caa	0.456665	-0.457925	0.582427	-0.584779	Putative disease resistance RPP13-like protein 1
AH12G02370	Chr12	2823268	C	A	C	24	24,0	1.00	M	6	1,5	0.17	0.83	NON_SYNONYMOUS_CODING	Ctg/Atg	0.456665	-0.457925	0.582427	-0.584779	Putative disease resistance RPP13-like protein 1
AH12G02390	Chr12	2845356	C	A	M	19	14,5	0.74	M	19	4,15	0.21	0.53	NON_SYNONYMOUS_CODING	Ctt/Att	0.456684	-0.457942	0.582449	-0.5848	Putative disease resistance RPP13-like protein 1
AH12G02390	Chr12	2845498	G	A	R	27	25,2	0.93	G	25	23,2	0.92	0.01	NON_SYNONYMOUS_CODING	cGc/cAc	0.456684	-0.457942	0.582449	-0.5848	Putative disease resistance RPP13-like protein 1
AH12G02390	Chr12	2845513	A	G	R	26	24,2	0.92	A	25	24,1	0.96	-0.04	NON_SYNONYMOUS_CODING	aAg/aGg	0.456684	-0.457942	0.582449	-0.5848	Putative disease resistance RPP13-like protein 1
AH12G02390	Chr12	2845530	T	C	Y	27	25,2	0.93	T	25	24,1	0.96	-0.03	NON_SYNONYMOUS_CODING	Tat/Cat	0.456684	-0.457942	0.582449	-0.5848	Putative disease resistance RPP13-like protein 1
AH12G02390	Chr12	2846684	G	T	G	22	22,0	1.00	K	18	10,8	0.56	0.44	NON_SYNONYMOUS_CODING	agG/agT	0.456684	-0.457942	0.582449	-0.5848	Putative disease resistance RPP13-like protein 1
AH12G02390	Chr12	2846808	A	G	A	17	17,0	1.00	R	25	13,12	0.52	0.48	NON_SYNONYMOUS_CODING	Aga/Gga	0.456684	-0.457942	0.582449	-0.5848	Putative disease resistance RPP13-like protein 1
AH12G02390	Chr12	2846850	T	A	T	15	15,0	1.00	W	26	15,11	0.58	0.42	NON_SYNONYMOUS_CODING	Tcc/Acc	0.456684	-0.457942	0.582449	-0.5848	Putative disease resistance RPP13-like protein 1
AH12G02390	Chr12	2846856	C	G	C	16	16,0	1.00	S	25	14,11	0.56	0.44	NON_SYNONYMOUS_CODING	Caa/Gaa	0.456684	-0.457942	0.582449	-0.5848	Putative disease resistance RPP13-like protein 1
AH12G02390	Chr12	2846949	A	G	A	11	11,0	1.00	R	15	12,3	0.80	0.20	NON_SYNONYMOUS_CODING	Aaa/Gaa	0.456684	-0.457942	0.582449	-0.5848	Putative disease resistance RPP13-like protein 1
AH12G02390	Chr12	2847652	G	T	G	15	14,1	0.93	K	6	2,4	0.33	0.60	NON_SYNONYMOUS_CODING	cGc/cTc	0.456684	-0.457942	0.582449	-0.5848	Putative disease resistance RPP13-like protein 1
AH12G02410	Chr12	2866827	A	G	A	21	21,0	1.00	R	4	1,3	0.25	0.75	NON_SYNONYMOUS_CODING	Aag/Gag	0.456704	-0.457961	0.582471	-0.584825	Putative disease resistance RPP13-like protein 1
AH12G02410	Chr12	2867664	G	C	G	18	17,1	0.94	C	19	1,18	0.05	0.89	NON_SYNONYMOUS_CODING	Gac/Cac	0.456704	-0.457961	0.582471	-0.584825	Putative disease resistance RPP13-like protein 1
AH12G02410	Chr12	2868121	C	G	S	25	21,4	0.84	S	17	2,15	0.12	0.72	NON_SYNONYMOUS_CODING	cCc/cGc	0.456704	-0.457961	0.582471	-0.584825	Putative disease resistance RPP13-like protein 1
AH12G02410	Chr12	2868129	C	G	S	27	22,5	0.81	S	17	3,14	0.18	0.64	NON_SYNONYMOUS_CODING	Caa/Gaa	0.456704	-0.457961	0.582471	-0.584825	Putative disease resistance RPP13-like protein 1
AH12G02520	Chr12	2952570	C	T	Y	24	21,3	0.88	Y	18	3,15	0.17	0.71	NON_SYNONYMOUS_CODING	Gga/Aga	0.456781	-0.458031	0.582557	-0.584916	1-aminocyclopropane-1-carboxylate synthase-like protein 1
AH12G02650	Chr12	3067519	T	C	Y	26	21,5	0.81	Y	12	2,10	0.17	0.64	NON_SYNONYMOUS_CODING	tTg/tCg	0.456889	-0.458138	0.582693	-0.585057	Reticulon-like protein B1
AH12G02880	Chr12	3276550	G	C	G	16	15,1	0.94	S	21	3,18	0.14	0.79	NON_SYNONYMOUS_CODING	tgG/tgC	0.457055	-0.458315	0.582914	-0.58527	Putative disease resistance RPP13-like protein 1
AH12G02960	Chr12	3347238	C	A	C	17	17,0	1.00	M	7	4,3	0.57	0.43	NON_SYNONYMOUS_CODING	Cgt/Agt	0.457096	-0.458361	0.582977	-0.585315	Transmembrane receptors 3BATP binding
AH12G02960	Chr12	3347384	T	A	T	27	25,2	0.93	W	8	4,4	0.50	0.43	NON_SYNONYMOUS_CODING	ttT/ttA	0.457096	-0.458361	0.582977	-0.585315	Transmembrane receptors 3BATP binding
AH12G02960	Chr12	3348195	C	G	C	5	5,0	1.00	S	10	1,9	0.10	0.90	NON_SYNONYMOUS_CODING	Cat/Gat	0.457096	-0.458361	0.582977	-0.585315	Transmembrane receptors 3BATP binding
AH12G03000	Chr12	3392506	C	G	C	24	24,0	1.00	S	9	2,7	0.22	0.78	NON_SYNONYMOUS_CODING	Ctt/Gtt	0.457119	-0.458386	0.583012	-0.585331	Protein of unknown function (DUF594)
AH12G03000	Chr12	3392515	C	A	C	22	21,1	0.95	M	9	2,7	0.22	0.73	NON_SYNONYMOUS_CODING	Ctg/Atg	0.457119	-0.458386	0.583012	-0.585331	Protein of unknown function (DUF595)
AH12G03230	Chr12	3693638	C	G	C	13	13,0	1	S	11	1,10	0.09	0.91	NON_SYNONYMOUS_CODING	aCt/aGt	0.457106	-0.4584	0.583117	-0.585359	putative disease resistance RPP13-like protein 1
AH12G03240	Chr12	3715337	G	C	G	13	13,0	1.00	S	22	4,18	0.18	0.82	NON_SYNONYMOUS_CODING	Gat/Cat	0.457108	-0.458403	0.583131	-0.585372	BAG family molecular chaperone regulator 6
AH12G03240	Chr12	3715406	T	C	T	17	17,0	1.00	Y	15	4,11	0.27	0.73	NON_SYNONYMOUS_CODING	Tgt/Cgt	0.457108	-0.458403	0.583131	-0.585372	BAG family molecular chaperone regulator 6
AH12G03240	Chr12	3715462	T	G	T	18	18,0	1.00	K	5	1,4	0.20	0.80	NON_SYNONYMOUS_CODING	atT/atG	0.457108	-0.458403	0.583131	-0.585372	BAG family molecular chaperone regulator 6
AH12G03290	Chr12	3745691	G	A	R	21	18,3	0.86	R	13	2,11	0.15	0.70	NON_SYNONYMOUS_CODING	cGt/cAt	0.457108	-0.458406	0.583152	-0.585391	Serine/threonine-protein phosphatase 7
AH12G03300	Chr12	3748516	C	T	Y	18	14,4	0.78	Y	20	4,16	0.20	0.58	NON_SYNONYMOUS_CODING	cGa/cAa	0.457108	-0.458406	0.583152	-0.585391	Aminotransferase-like 2C plant mobile domain family protein
AH12G03500	Chr12	3998818	G	A	G	20	20,0	1.00	R	14	5,9	0.36	0.64	NON_SYNONYMOUS_CODING	Ggt/Agt	0.456817	-0.45814	0.582958	-0.585158	MuDR family transposase
AH12G03500	Chr12	3999460	G	T	K	25	22,3	0.88	K	17	4,13	0.24	0.64	NON_SYNONYMOUS_CODING	aGg/aTg	0.456817	-0.45814	0.582958	-0.585158	MuDR family transposase
AH12G03500	Chr12	3999687	A	G	R	22	20,2	0.91	G	17	0,17	0.00	0.91	NON_SYNONYMOUS_CODING	Aac/Gac	0.46	-0.46	0.58	-0.59	MuDR family transposase
AH12G03560	Chr12	4096307	A	T	A	15	15,0	1.00	T	5	0,5	0.00	1.00	NON_SYNONYMOUS_CODING	gTg/gAg	0.46	-0.46	0.58	-0.59	UDP-glycosyltransferase 72B1
AH12G03600	Chr12	4142557	T	G	K	17	15,2	0.88	K	7	1,6	0.14	0.74	NON_SYNONYMOUS_CODING	Tct/Gct	0.46	-0.46	0.58	-0.59	Disease resistance protein TAO1
AH12G03600	Chr12	4142566	G	A	R	17	15,2	0.88	A	5	0,5	0.00	0.88	NON_SYNONYMOUS_CODING	Gtt/Att	0.46	-0.46	0.58	-0.59	Disease resistance protein TAO1
AH12G03600	Chr12	4142572	G	A	R	17	15,2	0.88	A	5	0,5	0.00	0.88	NON_SYNONYMOUS_CODING	Ggt/Agt	0.46	-0.46	0.58	-0.59	Disease resistance protein TAO1
AH12G03900	Chr12	4633134	C	T	Y	19	17,2	0.89	Y	15	2,13	0.13	0.76	NON_SYNONYMOUS_CODING	Gcg/Acg	0.46	-0.46	0.58	-0.58	Cytochrome b561 and DOMON domain-containing protein
AH12G03980	Chr12	4691788	C	T	Y	20	16,4	0.8	Y	17	4,13	0.24	0.56	STOP_GAINED	Caa/Taa	0.456446	-0.457759	0.582353	-0.585012	homolog of histone chaperone HIRA
AH12G03980	Chr12	4696605	G	A	G	25	25,0	1.00	R	12	2,10	0.17	0.83	NON_SYNONYMOUS_CODING	Gtt/Att	0.456446	-0.457759	0.582353	-0.585012	homolog of histone chaperone HIRA
AH12G04010	Chr12	4718691	G	T	K	21	16,5	0.76	T	16	1,15	0.06	0.70	NON_SYNONYMOUS_CODING	Ctg/Atg	0.456475	-0.457785	0.582373	-0.585056	Eukaryotic aspartyl protease family protein
AH12G04170	Chr12	5007281	T	A	T	23	23,0	1.00	W	22	19,3	0.86	0.14	NON_SYNONYMOUS_CODING	cAc/cTc	0.457503	-0.45874	0.58321	-0.586473	Endosomal targeting BRO1-like domain-containing protein
AH12G04170	Chr12	5007411	A	C	A	26	26,0	1.00	M	22	17,5	0.77	0.23	NON_SYNONYMOUS_CODING	Tca/Gca	0.457503	-0.45874	0.58321	-0.586473	Endosomal targeting BRO1-like domain-containing protein
AH12G04170	Chr12	5007427	G	C	G	27	27,0	1.00	S	22	17,5	0.77	0.23	NON_SYNONYMOUS_CODING	ttC/ttG	0.457503	-0.45874	0.58321	-0.586473	Endosomal targeting BRO1-like domain-containing protein
AH12G04170	Chr12	5007448	C	G	C	24	24,0	1.00	S	18	15,3	0.83	0.17	NON_SYNONYMOUS_CODING	tgG/tgC	0.457503	-0.45874	0.58321	-0.586473	Endosomal targeting BRO1-like domain-containing protein
AH12G04170	Chr12	5007454	C	G	C	27	27,0	1.00	S	17	14,3	0.82	0.18	NON_SYNONYMOUS_CODING	ttG/ttC	0.457503	-0.45874	0.58321	-0.586473	Endosomal targeting BRO1-like domain-containing protein
AH12G04330	Chr12	5187080	C	T	C	20	18,2	0.90	Y	10	3,7	0.30	0.60	NON_SYNONYMOUS_CODING	Gag/Aag	0.458456	-0.459622	0.584098	-0.587787	P-loop containing nucleoside triphosphate hydrolases superfamily protein
AH12G04490	Chr12	5526242	C	T	Y	25	19,6	0.76	Y	14	4,10	0.29	0.47	NON_SYNONYMOUS_CODING	gGa/gAa	0.460031	-0.461049	0.585491	-0.5901	Uncharacterized protein
AH12G04580	Chr12	5765500	G	T	G	18	17,1	0.94	K	6	1,5	0.17	0.78	NON_SYNONYMOUS_CODING	aaC/aaA	0.460793	-0.461707	0.58614	-0.591388	S-adenosyl-L-methionine-dependent methyltransferases superfamily protein
AH12G04670	Chr12	5864398	G	T	K	21	17,4	0.81	K	14	3,11	0.21	0.60	NON_SYNONYMOUS_CODING	Ggt/Tgt	0.46102	-0.461893	0.586301	-0.591789	Uncharacterized protein
AH12G04700	Chr12	5911414	G	A	G	18	17,1	0.94	R	13	1,12	0.08	0.87	NON_SYNONYMOUS_CODING	Gtg/Atg	0.461224	-0.462076	0.586503	-0.592101	Uncharacterized protein
AH12G04740	Chr12	6018424	T	C	Y	24	17,7	0.71	Y	16	5,11	0.31	0.40	NON_SYNONYMOUS_CODING	Tcc/Ccc	0.461787	-0.462593	0.587129	-0.59291	3-ketoacyl-CoA synthase 1
AH12G04980	Chr12	6321518	C	T	Y	22	17,5	0.77	Y	14	3,11	0.21	0.56	NON_SYNONYMOUS_CODING	cCc/cTc	0.462862	-0.46355	0.588336	-0.594617	Uncharacterized protein
AH12G05040	Chr12	6391830	A	G	R	19	15,4	0.79	R	18	3,15	0.17	0.62	NON_SYNONYMOUS_CODING	cAg/cGg	0.463056	-0.463716	0.588597	-0.594957	Pyruvate dehydrogenase E1 component subunit alpha-3/2C chloroplastic
AH12G05080	Chr12	6517918	T	C	T	12	12,0	1.00	Y	9	8,1	0.89	0.11	NON_SYNONYMOUS_CODING	Ttc/Ctc	0.463369	-0.463988	0.589041	-0.595523	Probably inactive leucine-rich repeat receptor-like protein kinase
AH12G05080	Chr12	6517937	A	T	A	14	14,0	1.00	W	12	10,2	0.83	0.17	NON_SYNONYMOUS_CODING	gAc/gTc	0.463369	-0.463988	0.589041	-0.595523	Probably inactive leucine-rich repeat receptor-like protein kinase
AH12G05080	Chr12	6517943	C	A	C	15	15,0	1.00	M	12	10,2	0.83	0.17	NON_SYNONYMOUS_CODING	tCt/tAt	0.463369	-0.463988	0.589041	-0.595523	Probably inactive leucine-rich repeat receptor-like protein kinase
AH12G05080	Chr12	6518088	T	A	T	18	18,0	1.00	W	7	5,2	0.71	0.29	NON_SYNONYMOUS_CODING	ttT/ttA	0.463369	-0.463988	0.589041	-0.595523	Probably inactive leucine-rich repeat receptor-like protein kinase
AH12G05100	Chr12	6594841	G	A	R	17	13,4	0.76	R	21	3,18	0.14	0.62	NON_SYNONYMOUS_CODING	gCg/gTg	0.463457	-0.464051	0.589192	-0.595747	Probably inactive leucine-rich repeat receptor-like protein kinase
AH12G05120	Chr12	6605230	G	A	R	22	17,5	0.77	A	13	0,13	0.00	0.77	NON_SYNONYMOUS_CODING	aCa/aTa	0.463458	-0.46405	0.589199	-0.595763	Flavone 3’-O-methyltransferase 1
AH12G05120	Chr12	6606476	T	A	T	20	20,0	1.00	W	13	10,3	0.77	0.23	NON_SYNONYMOUS_CODING	Atc/Ttc	0.463458	-0.46405	0.589199	-0.595763	Flavone 3’-O-methyltransferase 1
AH12G05120	Chr12	6607843	G	C	G	25	23,2	0.92	S	14	10,4	0.71	0.21	NON_SYNONYMOUS_CODING	aCt/aGt	0.463458	-0.46405	0.589199	-0.595763	Flavone 3’-O-methyltransferase 1
AH12G05140	Chr12	6655924	G	A	R	15	13,2	0.87	R	10	3,7	0.30	0.57	NON_SYNONYMOUS_CODING	gCt/gTt	0.463453	-0.464034	0.58922	-0.595823	Flavone 3’-O-methyltransferase 1
AH12G05250	Chr12	6843052	T	G	K	14	13,1	0.93	G	9	0,9	0.00	0.93	NON_SYNONYMOUS_CODING	Tcc/Gcc	0.463323	-0.463869	0.589187	-0.59586	B3 domain-containing mRNAion factor NGA1
AH12G05320	Chr12	6989102	A	G	R	10	9,1	0.90	R	12	2,10	0.17	0.73	NON_SYNONYMOUS_CODING	Tcc/Ccc	0.462905	-0.463448	0.588882	-0.5955	Serine/threonine-protein phosphatase 7
AH12G05420	Chr12	7082034	T	C	T	16	16,0	1.00	Y	15	1,14	0.07	0.93	NON_SYNONYMOUS_CODING	Aca/Gca	0.462547	-0.463075	0.588567	-0.595158	Probable inactive purple acid phosphatase 29
AH12G05820	Chr12	7671331	C	G	C	23	22,1	0.96	S	7	1,6	0.14	0.81	NON_SYNONYMOUS_CODING	caC/caG	0.460651	-0.461098	0.586887	-0.593277	FRIGIDA-like protein 3
AH12G05970	Chr12	7992939	G	A	R	12	11,1	0.92	R	15	2,13	0.13	0.78	NON_SYNONYMOUS_CODING	Cgc/Tgc	0.459751	-0.460171	0.585875	-0.592182	Aminotransferase-like 2C plant mobile domain family protein
AH12G06150	Chr12	8202579	C	T	T	21	0,21	1.00	Y	17	13,4	0.24	0.76	NON_SYNONYMOUS_CODING	Gaa/Aaa	0.459288	-0.459699	0.585408	-0.591564	AT-rich interactive domain-containing protein 4
AH12G06180	Chr12	8256014	C	A	C	21	20,1	0.95	M	21	3,18	0.14	0.81	NON_SYNONYMOUS_CODING	Cat/Aat	0.45923	-0.45964	0.585372	-0.591479	RING/U-box superfamily protein
AH12G06180	Chr12	8260084	G	A	G	22	22,0	1.00	R	9	6,3	0.67	0.33	NON_SYNONYMOUS_CODING	aGg/aAg	0.459218	-0.459627	0.585364	-0.591462	RING/U-box superfamily protein
AH12G06180	Chr12	8260159	T	C	T	16	16,0	1.00	Y	14	4,10	0.29	0.71	NON_SYNONYMOUS_CODING	tTt/tCt	0.459218	-0.459627	0.585364	-0.591462	RING/U-box superfamily protein
AH12G06180	Chr12	8260203	G	A	G	18	18,0	1.00	R	24	15,9	0.63	0.38	NON_SYNONYMOUS_CODING	Gtt/Att	0.459218	-0.459627	0.585364	-0.591462	RING/U-box superfamily protein
AH12G06180	Chr12	8271833	A	G	A	20	19,1	0.95	R	18	6,12	0.33	0.62	NON_SYNONYMOUS_CODING	tAt/tGt	0.459205	-0.459615	0.585355	-0.591445	RING/U-box superfamily protein
AH12G06180	Chr12	8271850	G	C	G	22	21,1	0.95	S	18	6,12	0.33	0.62	NON_SYNONYMOUS_CODING	Gca/Cca	0.459205	-0.459615	0.585355	-0.591445	RING/U-box superfamily protein
AH12G06210	Chr12	8303749	A	G	A	26	26,0	1.00	R	6	5,1	0.83	0.17	NON_SYNONYMOUS_CODING	Aca/Gca	0.459187	-0.459596	0.585344	-0.591416	RING/U-box superfamily protein
AH12G06210	Chr12	8303776	A	G	A	23	23,0	1.00	R	6	3,3	0.50	0.50	NON_SYNONYMOUS_CODING	Aaa/Gaa	0.459187	-0.459596	0.585344	-0.591416	RING/U-box superfamily protein
AH12G06250	Chr12	8416831	T	G	T	16	16,0	1.00	K	6	1,5	0.17	0.83	NON_SYNONYMOUS_CODING	Tgt/Ggt	0.459105	-0.459511	0.585247	-0.591281	RING/U-box superfamily protein
AH12G06300	Chr12	8578649	C	T	Y	16	13,3	0.81	Y	16	3,13	0.19	0.63	NON_SYNONYMOUS_CODING	gCt/gTt	0.458995	-0.459395	0.585101	-0.591125	Exocyst subunit exo70 family protein B2
AH12G06300	Chr12	8579347	C	T	C	12	12,0	1.00	Y	18	3,15	0.17	0.83	NON_SYNONYMOUS_CODING	Cgg/Tgg	0.458995	-0.459395	0.585101	-0.591125	Exocyst subunit exo70 family protein B2
AH12G06320	Chr12	8636540	G	T	G	18	18,0	1.00	T	9	0,9	0.00	1.00	NON_SYNONYMOUS_CODING	aaC/aaA	0.458954	-0.459351	0.585052	-0.591055	Putative disease resistance RPP13-like protein 1
AH12G06320	Chr12	8636563	C	A	C	18	18,0	1.00	A	8	0,8	0.00	1.00	NON_SYNONYMOUS_CODING	Gat/Tat	0.458954	-0.459351	0.585052	-0.591055	Putative disease resistance RPP13-like protein 1
AH12G06320	Chr12	8636575	G	A	G	13	13,0	1.00	A	7	0,7	0.00	1.00	NON_SYNONYMOUS_CODING	Cac/Tac	0.458954	-0.459351	0.585052	-0.591055	Putative disease resistance RPP13-like protein 1
AH12G06430	Chr12	8714663	C	A	M	12	11,1	0.92	M	19	3,16	0.16	0.76	NON_SYNONYMOUS_CODING	Cta/Ata	0.45886	-0.459253	0.584957	-0.590896	Duplicated homeodomain-like superfamily protein
AH12G06450	Chr12	8735310	C	T	C	14	14,0	1.00	Y	11	2,9	0.18	0.82	NON_SYNONYMOUS_CODING	aGa/aAa	0.458829	-0.459221	0.584923	-0.590847	Endosomal targeting BRO1-like domain-containing protein
AH12G06480	Chr12	8758727	G	T	G	16	15,1	0.94	K	9	4,5	0.44	0.49	NON_SYNONYMOUS_CODING	aCa/aAa	0.458799	-0.45919	0.58489	-0.5908	GDSL esterase/lipase 5
AH12G06480	Chr12	8767570	C	G	C	18	18,0	1.00	S	9	2,7	0.22	0.78	NON_SYNONYMOUS_CODING	caG/caC	0.458783	-0.459174	0.584873	-0.590776	GDSL esterase/lipase 5
AH12G06490	Chr12	8792582	T	G	T	21	21,0	1.00	K	13	7,6	0.54	0.46	NON_SYNONYMOUS_CODING	Tta/Gta	0.458733	-0.459123	0.584819	-0.590701	GDSL esterase/lipase 3
AH12G06560	Chr12	8898415	T	G	G	20	2,18	0.90	K	26	23,3	0.12	0.78	NON_SYNONYMOUS_CODING	gAt/gCt	0.458603	-0.458987	0.584691	-0.590474	UDP-glycosyltransferase 72B3
AH12G06950	Chr12	9324156	C	G	C	20	19,1	0.95	S	12	8,4	0.67	0.28	NON_SYNONYMOUS_CODING	tGg/tCg	0.458109	-0.458489	0.584069	-0.589328	Pentatricopeptide repeat-containing protein
AH12G06950	Chr12	9324470	T	C	T	16	15,1	0.94	Y	18	11,7	0.61	0.33	NON_SYNONYMOUS_CODING	aAt/aGt	0.458109	-0.458489	0.584069	-0.589328	Pentatricopeptide repeat-containing protein
AH12G07020	Chr12	9478950	G	A	R	8	1,7	0.88	R	6	5,1	0.17	0.71	NON_SYNONYMOUS_CODING	tGc/tAc	0.458059	-0.458444	0.583973	-0.589016	Squamosa promoter-binding-like protein 8
AH12G07090	Chr12	9663318	G	T	K	24	21,3	0.88	K	29	8,21	0.28	0.60	NON_SYNONYMOUS_CODING	Gca/Tca	0.457966	-0.458357	0.58382	-0.588646	mRNAion regulators

**Table 3:** Identification of InDels in putative candidate genes in the genomic region for resistance to on chromosome 12.

Gene	Chromosome	Physical position (bp)	Reference Genome	Alternative site	Resistant bulk (RB) base	Number of reads covering the site (X coverage) in resistant bulk (RB)	RB Depths of Ref, Alt	SNP-index of RB	Susceptible bulk(SB)base	Number of reads covering the site (X coverage) in susceptible bulk(SB)	SB Depths of Ref, Alt	SNP-index of SB	delta SNP-index (RB SNP-index-SB SNP-index)	SNP substitution effect	U95 (95% confidence interval upper side)	L95 (95% confidence interval lower side)	U99 (99% confidence interval upper side)	L99 (99% confidence interval lower side)	Gene Function
AH12G01550	Chr12	1888286	TCC	T	TCC	23	22,1	0.96	TCC,T	9	7,2	0.78	0.18	FRAME_SHIFT	0.499429	-0.498581	0.630045	-0.613463	putative disease resistance protein At3g14460 isoform X2 [Arachis ipaensis]
AH12G01670	Chr12	2019127	CCTATTCTACG CCTCACAAAATCT	C	CCTATTCTACG CCTCACAAAATCT	28	26,2	0.93	CCTATTCTACGCCTCACAAAATCT,C	22	17,5	0.77	0.16	FRAME_SHIFT	0.499034	-0.498174	0.629563	-0.613179	HXXXD-type acyl-transferase family protein
AH12G01670	Chr12	2020084	T	TGGTGAAACA	T	21	21,0	1.00	T,TGGTGAAACA	15	10,5	0.67	0.33	CODON_INSERTION	0.498917	-0.498054	0.629416	-0.613083	HXXXD-type acyl-transferase family protein
AH12G01920	Chr12	2473096	A	ACGTTT	A,ACGTTT	22	19,3	0.86	A,ACGTTT	16	2,14	0.13	0.74	FRAME_SHIFT	0.496175	-0.495264	0.626054	-0.611224	Putative disease resistance protein At3g14460
AH12G01980	Chr12	2534345	CTCCGTACCAAG	C	C,CTCCGTACCAAG	24	22,2	0.92	C,CTCCGTACCAAG	20	18,2	0.90	0.02	FRAME_SHIFT	0.495902	-0.494987	0.625693	-0.611031	Putative disease resistance RPP13-like protein 1
AH12G01980	Chr12	2539604	TA	T	TA	24	24,0	1.00	TA,T	20	17,3	0.85	0.15	FRAME_SHIFT	0.495902	-0.494987	0.625693	-0.611031	Putative disease resistance RPP13-like protein 1
AH12G01980	Chr12	2539716	C	CCA	C	25	25,0	1.00	CCA,C	25	15,10	0.60	0.40	FRAME_SHIFT	0.495902	-0.494987	0.625693	-0.611031	Putative disease resistance RPP13-like protein 1
AH12G02090	Chr12	2620516	TGG	T	TGG	24	24,0	1.00	T,TGG	9	8,1	0.89	0.11	FRAME_SHIFT	0.495698	-0.494779	0.625463	-0.610928	Putative disease resistance RPP13-like protein 1
AH12G02390	Chr12	2847658	T	TCAAA	T	16	15,1	0.94	T,TCAAA	6	2,4	0.33	0.60	FRAME_SHIFT	0.495495	-0.494571	0.625259	-0.610874	Putative disease resistance RPP13-like protein 1
AH12G02440	Chr12	2879448	T	TGAG	T	11	11,0	1.00	T,TGAG	6	5,1	0.83	0.17	CODON_CHANGE_PLUS_CODON_INSERTION	0.495507	-0.494586	0.625287	-0.610914	Putative disease resistance RPP13-like protein 1
AH12G02600	Chr12	3030085	T	TGATGGTGAGA CTTTGAAGAGCA ACCAGTCCTTC TCTTGGAAATGAC ACAAATGAGTTGCAGTGATG	TGATGGTGAGACTTT GAAGAGCAACCAGTC CTTCTCTTGGAAATGA CACAAATGAGTTGCAGTGATG,T	8	7,1	0.88	T,TGATGGTGAG ACTTTGAAGAGCA ACCAGTCCTTCTC TTGGAAATGACAC AAATGAGTTGCAGTGATG	13	2,11	0.15	0.72	CODON_INSERTION	0.495575	-0.494661	0.625474	-0.61112	Putative disease resistance RPP13-like protein 1
AH12G03240	Chr12	3715392	ATCG	A	ATCG	17	17,0	1.00	A,ATCG	17	4,13	0.24	0.76	CODON_DELETION	0.496363	-0.495453	0.627006	-0.612884	BAG family molecular chaperone regulator 6
AH12G06110	Chr12	8165877	G	GGATTAGTGGTGCAGCAGCTTGT	G	20	20,0	1.00	G,GGATTAGTGGTGCAGCAGCTTGT	10	3,7	0.30	0.70	FRAME_SHIFT	0.502752	-0.502787	0.64146	-0.630466	hypothetical protein MTR_5g086360
AH12G06180	Chr12	8289562	CTAA	C	CTAA	10	10,0	1.00	CTAA,C	21	19,2	0.90	0.10	CODON_DELETION	0.502481	-0.502506	0.641028	-0.630118	E3 ubiquitin-protein ligase RNF144A-like

### 3.6 Allele-specific marker development and validation

**Figure 5:**
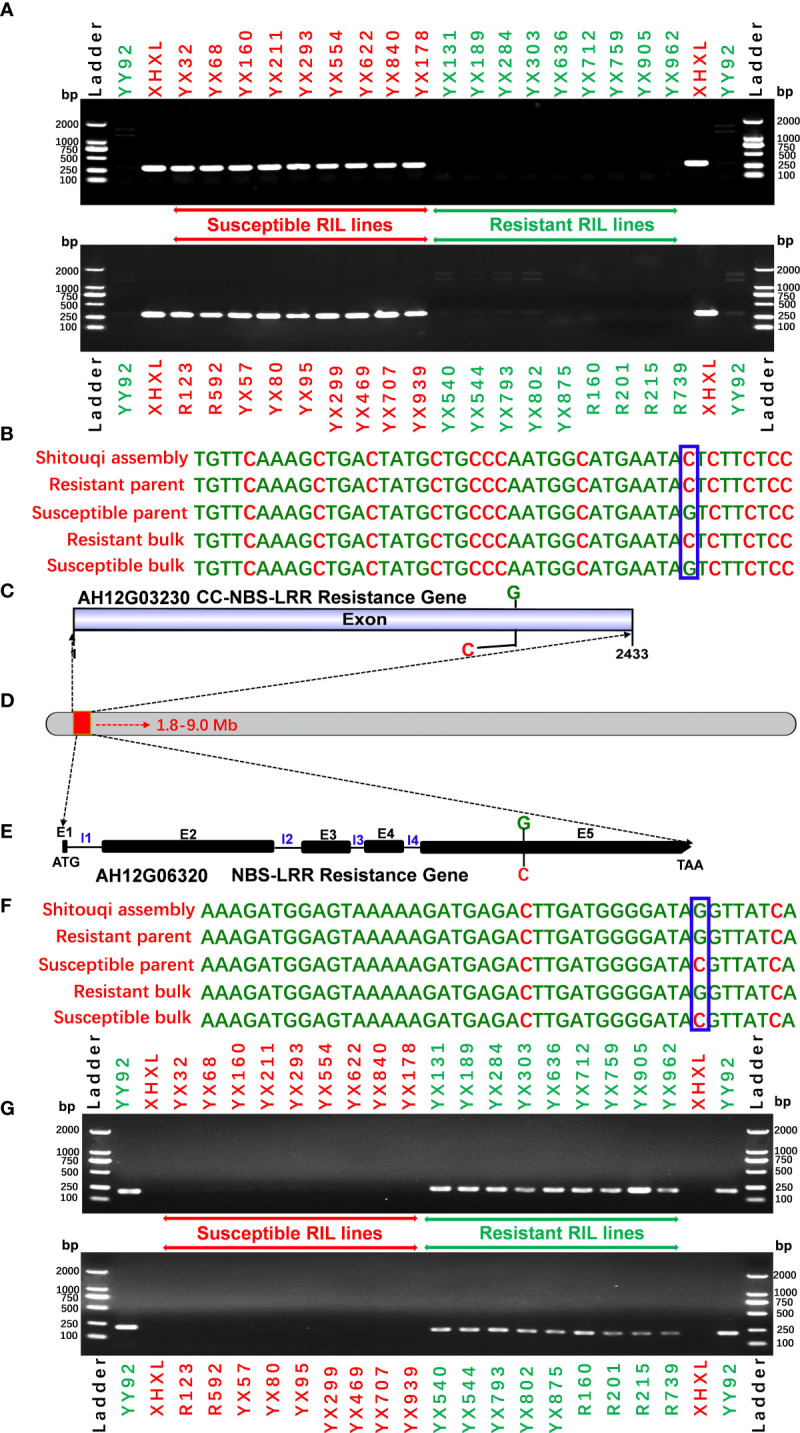
Validation of putative candidate gene-based markers of bacterial wilt resistance. **(A)** The SNP marker validation of candidate gene *AH12G03230* using a validation set comprising the resistant parent YY92, susceptible parent XHXL, and susceptible and resistant RIL lines). **(B)** SNP variation in the *AH12G03230* gene. **(C)**The *AH12G03230* gene is predicted to encode the CC-NBS-LRR resistance protein. **(D)** Putative genomic region on Chromosome 12 of *Arachis hypogaea* that encodes resistance to *Ralstonia solanacearum* infections **(E)** The *AH12G06320* gene is predicted to encode the NBS-LRR resistant protein (E1 to E5 refer to exon numbers while I1 to I4 refer to intron numbers), **(F)** SNP variation in the *AH12G06000* gene and **(G)** marker validation on a validation set comprising resistant parent YY92, susceptible parent XHXL, susceptible RIL lines (YX32, YX68, YX160, YX211, YX293, YX554, YX622, YX840 and YX178, R123, R592, YX57, YX80, YX95, YX299, YX469, YX707 andYX939), and resistant RIL lines (YX131, YX189, YX284, YX303, YX636, YX712, YX759, YX905, YX962, YX540, YX544, YX793, YX802, YX875, R160, R201, R215 and R739).

To evaluate the specificity of the allele marker of the resistant and susceptible peanut cultivars, we targeted 44 SNPs from candidate NBS-LRR genes for RRS for the development of allele-specific markers (**Supplementary Table 6**). Allele-specific primers for 44 SNPs were successfully generated. All 44 allele-specific markers were checked for polymorphisms between parental genotypes of the RIL population (YY92 and XHXL). Of the 44 markers, 30 allele markers had good amplification, whereas 14 markers did not yield amplicons with clear bands from samples of parental genotypes. Of the 30 amplified markers, two markers (*qRRS18* and *qRRS19*) in *AH12G03230* and *AH12G06320* genes co-segregated with RRS and may thus be deployed for RRS breeding (**Figure 5**). These two polymorphic markers were validated on a panel of diverse genotypes containing the resistant parent (Yueyou 92), 18 introgression-resistant RIL lines (YX131, YX189, YX284, YX303, YX636, YX712, YX759, YX905, YX962, YX540, YX544, YX793, YX802, YX875, R160, R201, R215 and R739), the susceptible parent (Xinhuixiaoli), and 18 susceptible RIL lines (YX32, YX68, YX160, YX211, YX293, YX554, YX622, YX840, YX178, R123, R592, YX57, YX80, YX95, YX299, YX469, YX707 and YX939). The primers for the diagnostic marker ‘*qRRS18*’ amplified a 302-bp fragment in the susceptible parent and different susceptible RIL lines, but none in the resistant genotypes (**Figure 5A**). In contrast, primers for another diagnostic marker ‘*qRRS19*’ amplified 217-bp fragment in the resistant genotypes and none in susceptible lines (**Figure 5G**). Most importantly, these two diagnostic markers (*qRRS18* + *qRRS19*) could be further developed and used to distinguish between homozygotes and heterozygotes in the segregating population; i.e., susceptible lines will have a 302-bp allele from the marker ‘*qRRS18*’ and resistant lines will have a 217-bp allele from the marker ‘*qRRS19*’. These two markers can be used as diagnostic marks for breeding resistant bacterial wilt varieties *via* MAS approach.

## 4 Discussion

With the advent of complete genome sequencing of diploid progenitor species and cultivated tetraploid variants, the QTL-seq approach is an increasingly popular sequencing-based method for the identification of candidate genomic regions associated with target traits in peanut (Varshney et al., 2019; Pandey et al., 2020). As it only requires whole genome sequences of parents and extreme trait bulks from the mapping population, it is economical, efficient, fast, and cost-effective (Takagi et al., 2013). Traditional QTL mapping methods are limited in the fine mapping of target genes and QTLs because they lack both high-density genetic maps and a series of near-isogenic lines (Pandey et al., 2017). Despite not having a large segregation population as a prerequisite, the QTL-seq approach was successful in identifying candidate genes for many crop traits (Das et al., 2014; Lu et al., 2014; Illa et al., 2015; Singh et al., 2016; Wang et al., 2016; Pandey et al., 2017; Srivastava et al., 2017; Chen et al., 2018; Clevenger et al., 2018; Luo et al., 2019; Bommisetty et al., 2020; Kumar et al., 2020; Lei et al., 2020; Tudor et al., 2020; Zhao et al., 2020; Cao et al., 2021; Dong et al., 2021; Topcu et al., 2021; Wang et al., 2021; Yang et al., 2021; Zhang et al., 2021). In the present study, a QTL-seq approach was successfully applied to identify genomic regions and candidate genes for RRS using resequencing data of both parental genotypes and pooled samples of the RIL population (Yueyou 92×Xinhuixiaoli) (**Supplementary Figure 1**), which corroborated our previously reported QTL mapping findings (Zhao et al., 2016).

The use of a common reference genome that is associated with deep sequencing and large bulks should result in highly accurate maps. As per the original QTL-seq approach (Takagi et al., 2013), the genome assemblies of either one or both parents were used as reference to analyze the SNP variants in the two extreme bulks based on diploid reference genomes due to the unavailability of the cultivated peanut genome (Pandey et al., 2017; Luo et al., 2019; Kumar et al., 2020; Chen et al., 2021). Candidate genomic regions were then associated with target traits by the ΔSNP index method (Takagi et al., 2013). The choice of the parental reference genome possibly affects this association of candidate genomic regions due to differing levels of alignment errors (Luo et al., 2019). Moreover, the algorithm uses the reference genomes to replace the parental genomes in bigger diversity areas, which may cause erroneous assemblies of the parent genomes, especially for wild diploid ancestors (unpublished data). To increase the reliability of identified genomic regions and candidate genes, we used the *Arachis hypogaea* Shitouqi genome as a reference. Shitouqi (*A. h. fastigiata* var. *vulgaris*), belongs to the subsp. *fastigiata*, as does the parents of the population, and its high-quality genome sequence was recently reported (Zhuang et al., 2019). Based on a large RIL population of 581 individual lines from the cross of resistant YY92 and susceptible Xinhuixiaoli, 30 resistant and 30 susceptible lines were selected to respectively construct the extreme R and S-bulks (**Figure 1** and **Supplementary Table 1**). We generated 108.38 and 96.92 Gb of sequence reads at a sequencing depth of 38× and 34× for the R- and S-bulks, respectively (**Table 1**). Nearly 98% of these reads were correctly mapped onto the reference genome. High densities of homozygous SNPs (381,642) and InDels (98,918) between parents were identified by resequencing for RRS mapping (**Supplementary Tables 2, 3**). The candidate region of 5.73 Mb on Chr12 was identified by combining the ED and ΔSNP/InDel index algorithms for both SNPs and InDels (**Figures 2**, **5**; **Table 2**; **Supplementary Tables 2, 3**) at a *P*<0.01 confidence level. These aided the precise and accurate discovery of candidate genomic regions, genes, and SNPs/Indels markers associated with RRS.

The clear extreme phenotypic differences between parents as well as those of the pooled population were the crucial prerequisite for candidate gene mapping *via* the QTL-seq approach (Zhao et al., 2020; Chen et al., 2021). An R2R3-MYB transcription factor gene named *AhTc1* was mapped and characterized as associated with purple testa *via* the QTL-seq approach. Allele-specific markers were developed, which demonstrated that the marker *pTesta1089* was closely linked with purple testa (Zhao et al., 2020). Chen et al. identified *AhRt1* bHLH transcriptional factor as the candidate gene that regulates the red testa color of peanut *via* QTL-sequencing analyses. An *AhRt1* diagnostic marker was then developed for validating and distinguishing different populations and peanut varieties (Chen et al., 2021). The phenotype evaluation of plant disease resistance traits is a challenge for map-based gene cloning. Unlike the testa color phenotype, disease resistance traits are complicated and affected by the environment, especially those for resistance to peanut bacterial wilt. As usual, the identification method in the disease nursery was used for the resistant evaluation. The survival rate was calculated from the number of dead plants until the point of harvesting for QTL mapping of BWR (Luo et al., 2019). However, the natural identification method was affected by the temperature, the soil environment, and anthropogenic effects. In our previous study, artificial inoculation by the leaf-cutting method was successfully used to evaluate the resistance to bacterial wilt *via* high throughput sequencing for QTL mapping of the cultivated peanut (Zhao et al., 2016). The resistance phenotyping in this study validated the accuracy of our previous findings (Zhao et al., 2016). The disease symptoms were classified into six disease severity ratings, and the resistance level of different lines was calculated by the disease index (**Figure 1** and **Supplementary Table 1**). The phenotype of different lines was truly reflective of the disease resistance as per the DI method and the candidate genomic region was then accurately identified by the QTL-seq approach (**Figure 2** and **Supplementary Figures 6**-**9**). This method is clearly valuable in the phenotyping of RRS for large populations in a cost-effective and practicable manner.

Hitherto, the main stable QTLs of RRS in the peanut were successfully identified through the original QTL method and QTL-seq approach (Luo et al., 2020). We previously reported SSR and SNP marker-based genetic linkage maps obtained through the classical QTL mapping (Zhao et al., 2016). Two major QTLs (*qBW-1* and *qBW-2*) were identified for RRS, which were in the LG1 and LG10 linkage groups based on RAD- and BSA-seq techniques in F_2_ plants. One QTL linked to two QTL peaks on ChrB02 was identified in an F_8_ RIL population (Zhao et al., 2016). Luo et al. reported one QTL named *qBWRB02.1* that possibly spanned a 5.14 Mb (0.81–5.95 Mb) interval on chromosome B02 based on its flanking SSR markers (Luo et al., 2020). *Via* the QTL-seq approach, they then identified a 2.07 Mb genomic region on ChrB02 associated with RRS across three environments (Luo et al., 2020). Two adjacent genomic regions (2.81–4.24 Mb and 6.54–8.75 Mb) on chromosome B02 were identified within the confidential interval of *qBWRB02-1* and thus designated as *qBWRB02-1-1* and *qBWRB02-1-2* based on two diploids reference genomes (Luo et al., 2019). In the present study, by using the QTL-seq approach (**Supplementary Figure 1**), a major peak on Chr12 spanning a 7.2 Mb (1.8–9.0 Mb) interval with a confidence level of *P*<0.05, 5.73 Mb of this peak had a confidence level of *P*<0.01 was identified as the candidate genomic region for RRS, corroborating RAD-seq findings (Zhao et al., 2016) and SLAF-seq techniques (**Figure 3** and **Supplementary Figure 10**, unpublished). These revealed the precise identification of the candidate genomic region *via* the QTL-seq approach. In China, peanut cultivars that are resistant to bacterial wilt, originate from Xiekangqing, Taishan Zhenzhu, and the wildtype species (*A.diogoi*). Two major QTLs that were both named *qBWRB02.1*, were identified from the cross of Yuanza 9102 × Xuzhou 68-4. Yuanza 9102 is a popular resistant cultivar whose resistance stemmed from *A.diogoi* (Luo et al., 2020). Two major QTLs, *qBWRB02-1-1* and *qBWRB02-1-2*, were fine-mapped from the cross of Zhonghua 6 × Xuhua 13. The resistance phenotype of Zhonghua 6 stemmed from the Chinese landrace Taishan Zhenzhu (Luo et al., 2019) whereas that of Yueyou92 stemmed from the Chinese landrace Xiekangqing, which is the parental type for many RRS breeding programs in South China (Janila et al., 2016; Luo et al., 2020). These candidate genomic regions associated with RRS were also mapped onto an interval of 10 Mb on chromosome 12.

QTL-seq approach was demonstrated as an effective method for the identification of putative SNPs associated with RRS. These could be developed into allele markers by using either different genotypes or diagnostic markers after the validation (Luo et al., 2019). Allele-specific markers that can be identified *via* agarose gel electrophoresis are the most cost-effective assays for genotyping a breeding population to select plants with the desired allele (Pandey et al., 2017). Here, 1807 effective SNPs and 629 InDels were identified. They span a 7.2 Mb genomic region on chromosome 12 that is associated with RRS. A total of 180 nonsynonymous SNPs and 14 InDels respectively affected 75 and 11 candidate genes that encode RRS (**Tables 2**, **3**, **Supplementary Tables 4** and **5**). The putative RRS-encoding NBS-LRR gene had 44 SNPs, which were targeted for the development of allele-specific markers. Despite designing primers for both alleles of each SNP, amplification of markers was often observed for only one of the allele pairs. Nonamplification of a few markers may be due to DNA template-primer mismatches (You et al., 2008; Pandey et al., 2017). In this study, polymorphic SNP markers were selected as diagnostic markers. Of the 30 amplified markers, two markers (*qRRS18* and *qRRS19*) were robust and co-segregated with RRS (**Figure 5**). These two polymorphic markers were then validated on a panel of diverse genotypes containing naturally resistant parental types (Yueyou 92) of the RIL population, 18 introgression lines, susceptible parental types (Xinhuixiaoli), and 18 susceptible RIL lines. The ‘*qRRS18*’ marker amplified susceptible alleles, whereas the ‘*qRRS19*’ marker amplified resistant alleles. Thus, these diagnostic allele markers can be applied in MAS for RRS in peanut breeding programs.

In plants, *R* genes play an important role in defending against pathogens through activation of the innate immune system (Jones and Dangl, 2006; Zhang et al., 2017; Sun et al., 2020; Chang et al., 2022). Most of them belong to the NBS-LRR type, which has been identified in many crops by map-based cloning (Takken and Joosten, 2000; Hulbert et al., 2001; McDowell and Woffenden, 2003; Meyers et al., 2005). The function of the NBS-LRR genes was correlated with either protein length or SNP variants. *RRS1-R* was the first reported Tir-NBS-LRR gene that conferred resistance to bacterial wilt in *Arabidopsis* species (Deslandes et al., 2003). *RRS1-S*, the allele of *RRS1-R* found in susceptible species, encode a protein without the WRKY domain. The two genes encoded contrasting phenotypes after *R. solanacearum* infections (Deslandes et al., 1998; Deslandes et al., 2003). Deng et al. identified an NBS-LRR gene named *PigmR*. It conferred resistance to the fungus *Magnaporthe oryzae* in rice and its encoded protein lacked four amino acids in the leucine-rich repeat (LRR) domain when compared to the *R4* gene that conferred a susceptible phenotype (Deng et al., 2017). In the present study, the predicted products of 22 of the 180 candidate genes with nonsynonymous mutations in the 7.2 Mb region, were NBS-LRR type disease resistance proteins (**Table 2** and **Figure 4**). Notably, eight of the 22 candidate NBS-LRR genes were identified at a high confidence level as associated with RRS (**Figure 4**). Moreover, seven NBS-LRR genes had SNP variant sites in the LRR domain (**Figure 4**). The diagnostic SNP markers (*qRRS18* and *qRRS19*) of the candidate *AH12G03230* and *AH12G06320* NBS-LRR genes were validated in the RIL lines (**Figure 5**). This indicates that *AH12G03230* and *AH12G06320* might be the candidate resistant genes for RRS in cultivated peanut. Therefore, based on these findings, these putative resistance genes possibly significantly contribute to RRS in peanut and should thus be targeted as candidates for fine mapping and function validation.

## 5 Conclusion

In this study, the QTL-seq approach was proven as a powerful method for the successful identification of genomic regions and candidate genes of major and robust QTLs that are associated with RRS. We not only identified a 7.2 Mb genomic region on chromosome 12 containing eight candidate NBS-LRR resistance genes but also availed validated allele-specific diagnostic markers and key candidate genes for RRS breeding. The genomic information (genes) and tools (markers) could be used in genomics-assisted breeding programs to accelerate the development of peanut varieties with enhanced RRS as well as to increase insights into RRS molecular mechanisms.

## Data availability statement

The original contributions presented in the study are publicly available. This data can be found here: NCBI, PRJNA851221.

## Author contributions

WZ and RV conceived the original research plan and designed the experiment. CZ, WX, HF, YC, HC, TC, QY, YZ, KC, and XZ performed the experiments and analyzed the data. CZ and WX wrote the manuscript, while WZ, MP, and RV reviewed and edit the manuscript. All authors analyzed the data and approved the submitted version.
