# Supplementary material for: Whole genome resequencing identifies candidate genes and allelic diagnostic markers for resistance to Ralstonia solanacearum infection in cultivated peanut (Arachis hypogaea L.)
Source: Front Plant Sci. 2023 Jan 4;13:1048168. doi: 10.3389/fpls.2022.1048168 (PMC9845939; doi:10.3389/fpls.2022.1048168)
Supplement: Supplementary file 1 [file DataSheet_1.pdf]

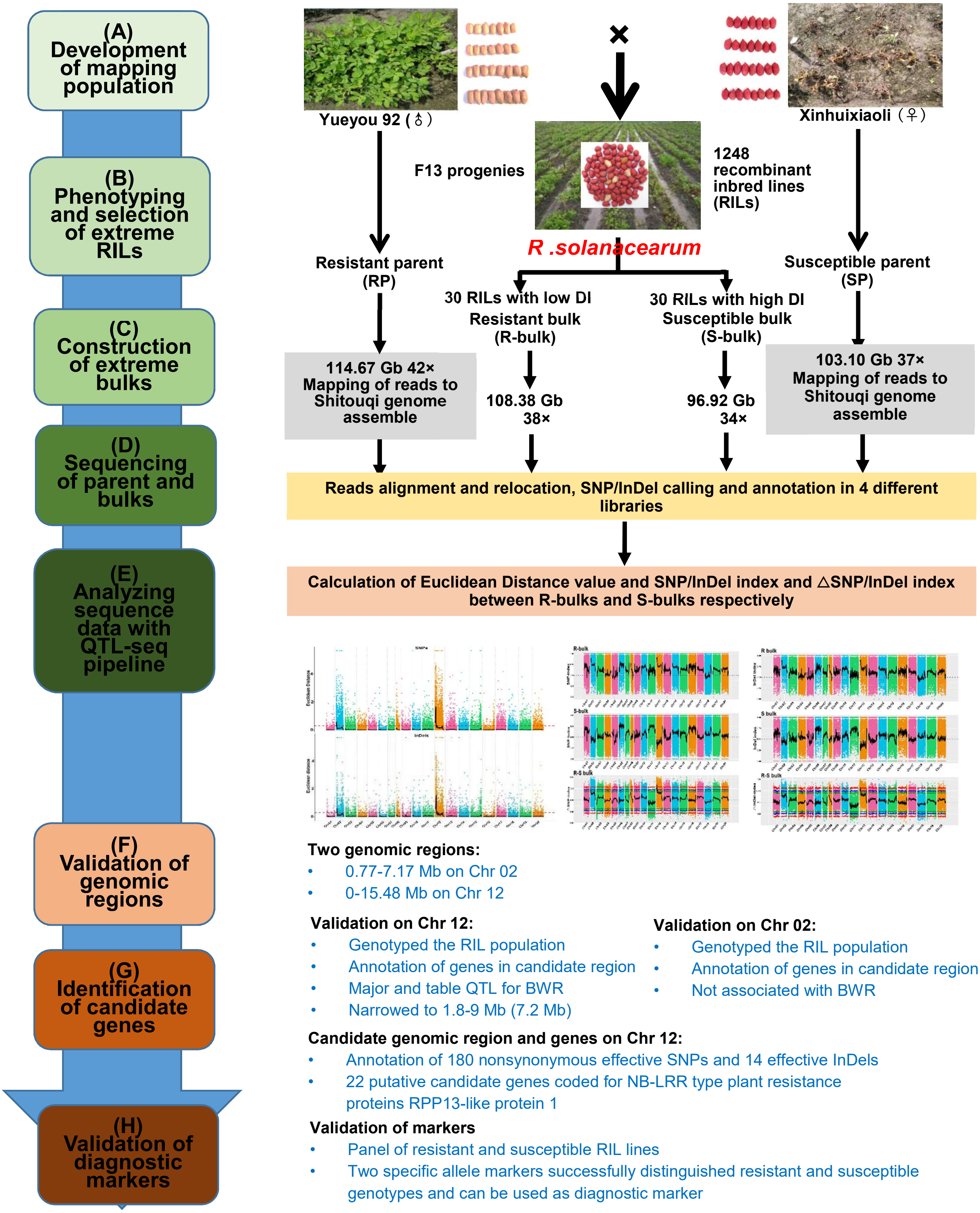

Supplementary Figure 1 QTL-seq approach used for mapping bacterial wilt resistance in cultivated peanut.

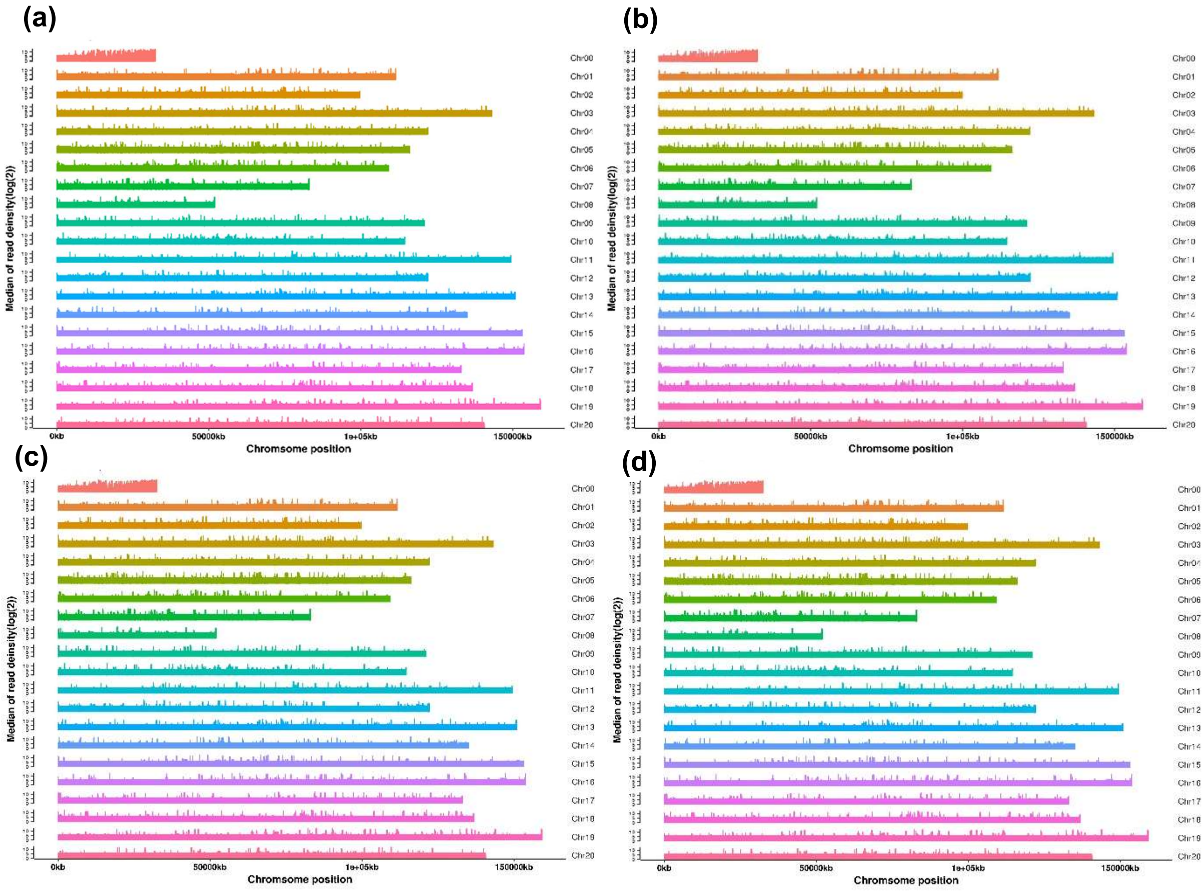

Supplementary Figure 2 Genome wide distribution of reads coverage in different 4 samples. The *X*-axis represents the chromosome position, and the *Y*-axis represents the coverage depth of the corresponding position on the chromosome, which is obtained by taking logarithm (log2). a, b, c, d was the reads coverage distribution in the genome of YY92, XHXL, R-bulk and S-bulk, respectively.

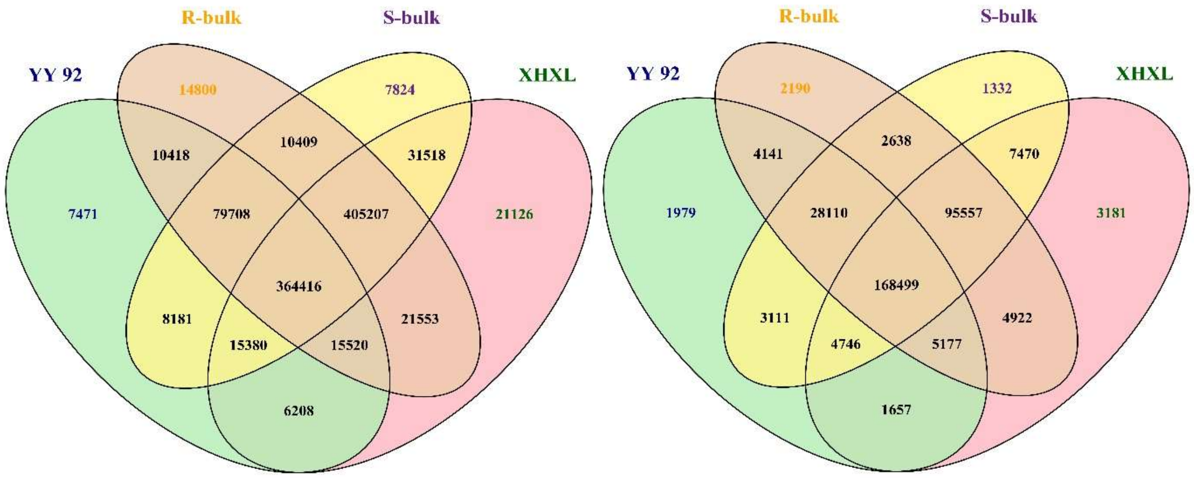

Supplementary Figure 3 Venn diagram of SNP and InDels information in different 4 samples.

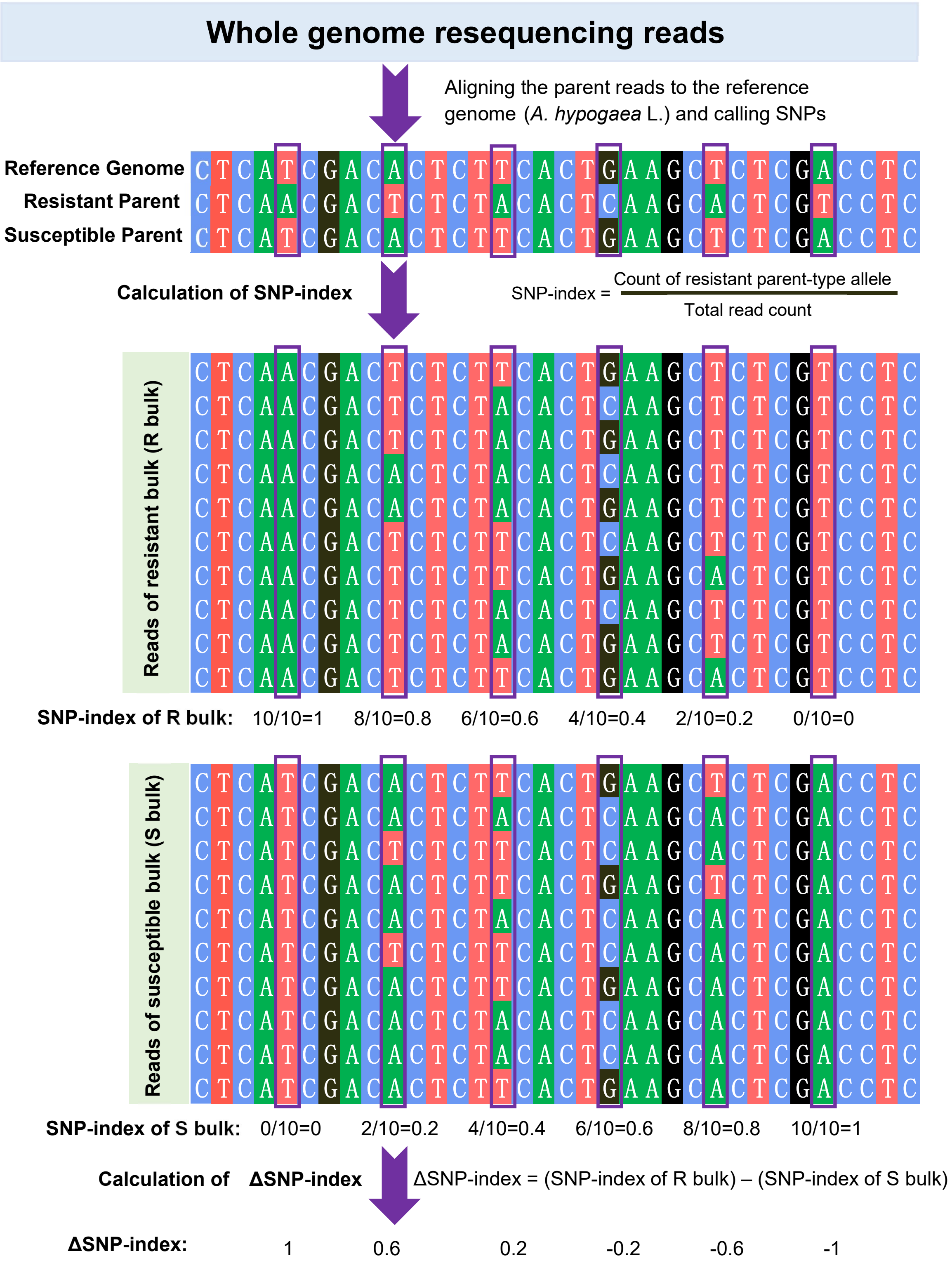

Supplementary Figure 4 Alignment, SNP identification and calculation of SNP-index and ΔSNP-index for resistance to *Rastonia solanacearum* infection.

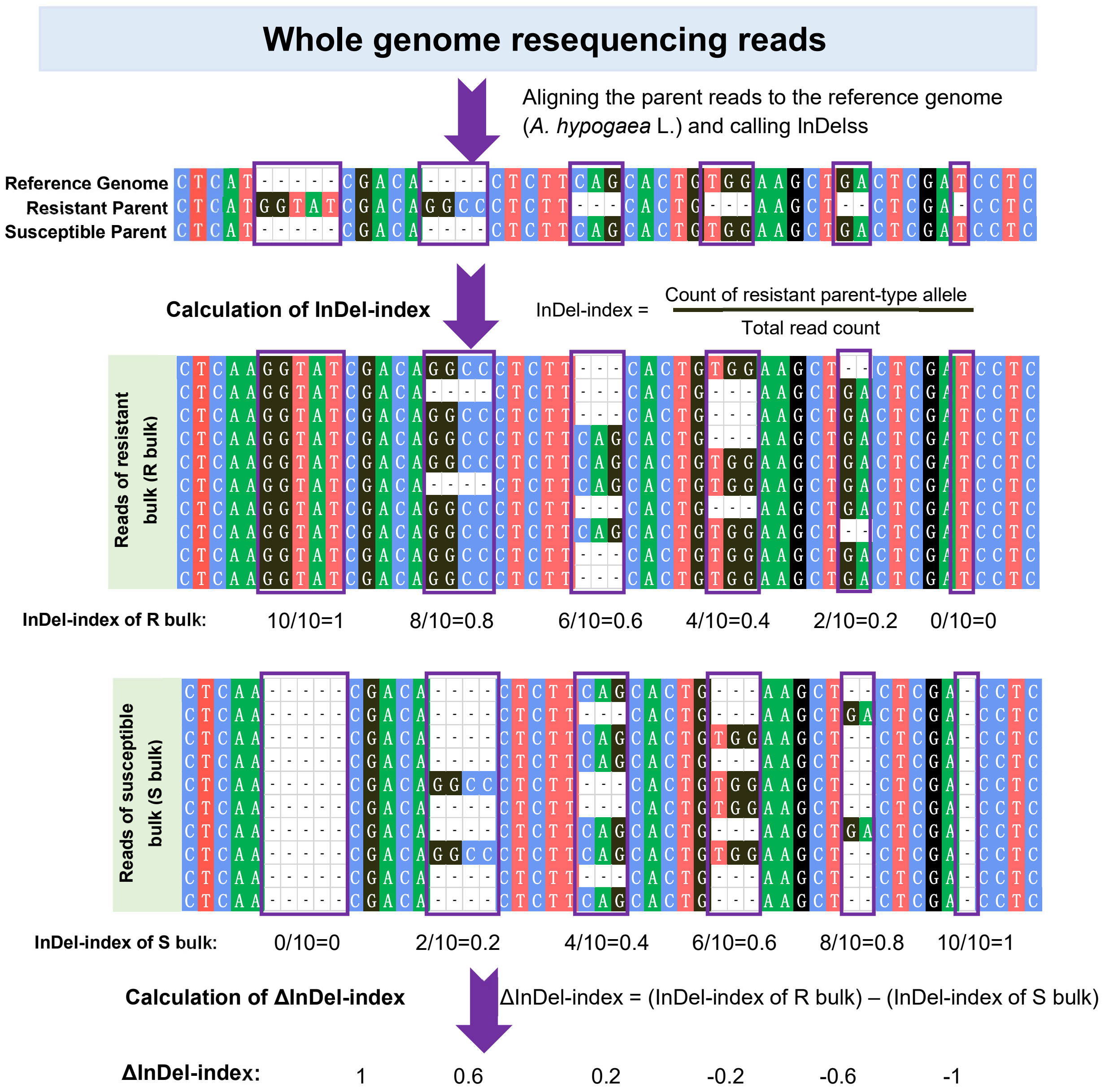

Supplementary Figure 5 Alignment, InDel identification and calculation of InDel-index and ΔInDel-index for resistance to *Rastonia solanacearum* infection.

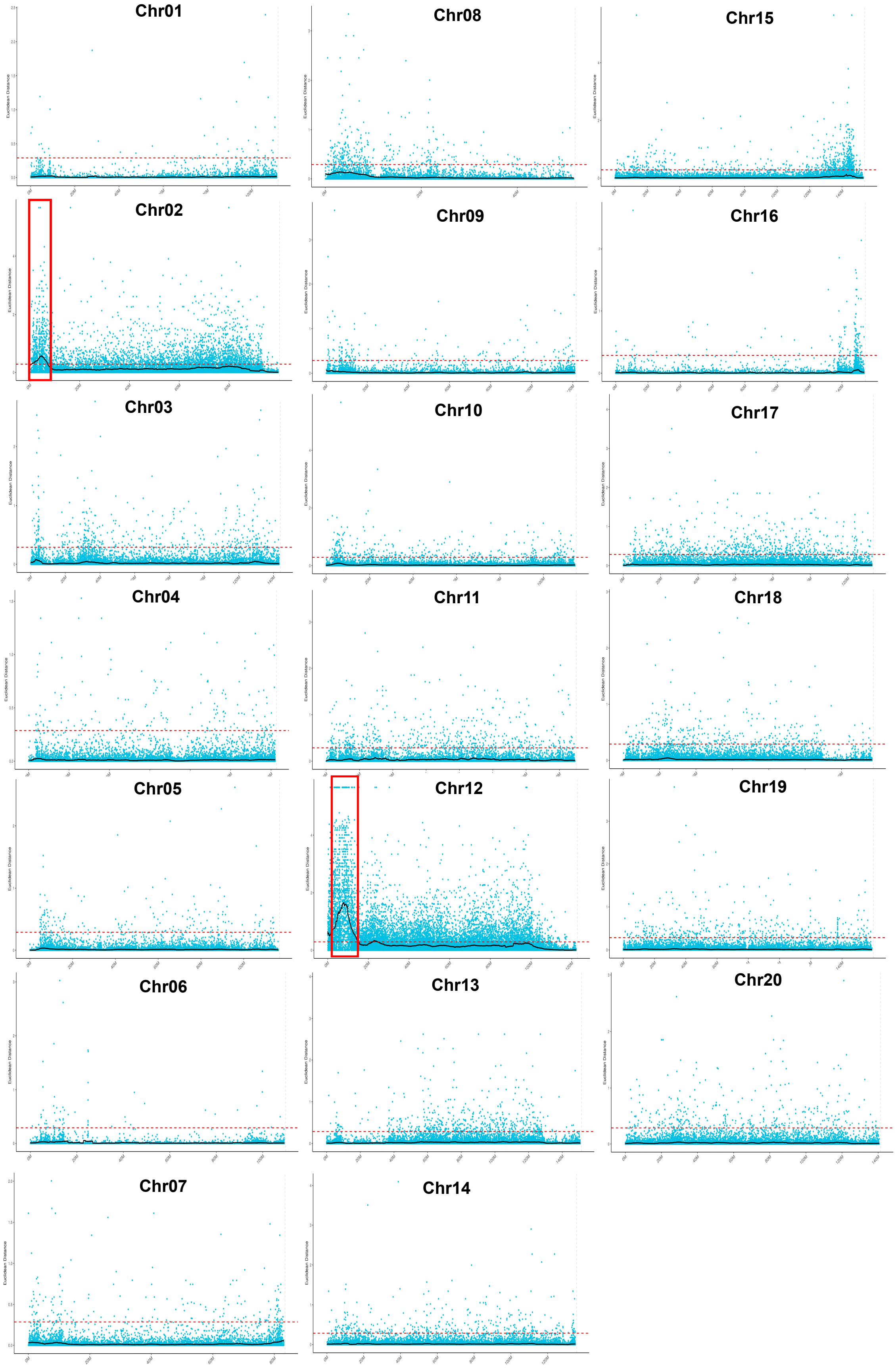

Supplementary Figure 6 Euclidean distance (ED) value distribution of SNPs for different 20 chromosomes for bacterial wilt resistance. Red lines indicate the sliding window average of 2 Mb interval with 100 kb increment for ED value.

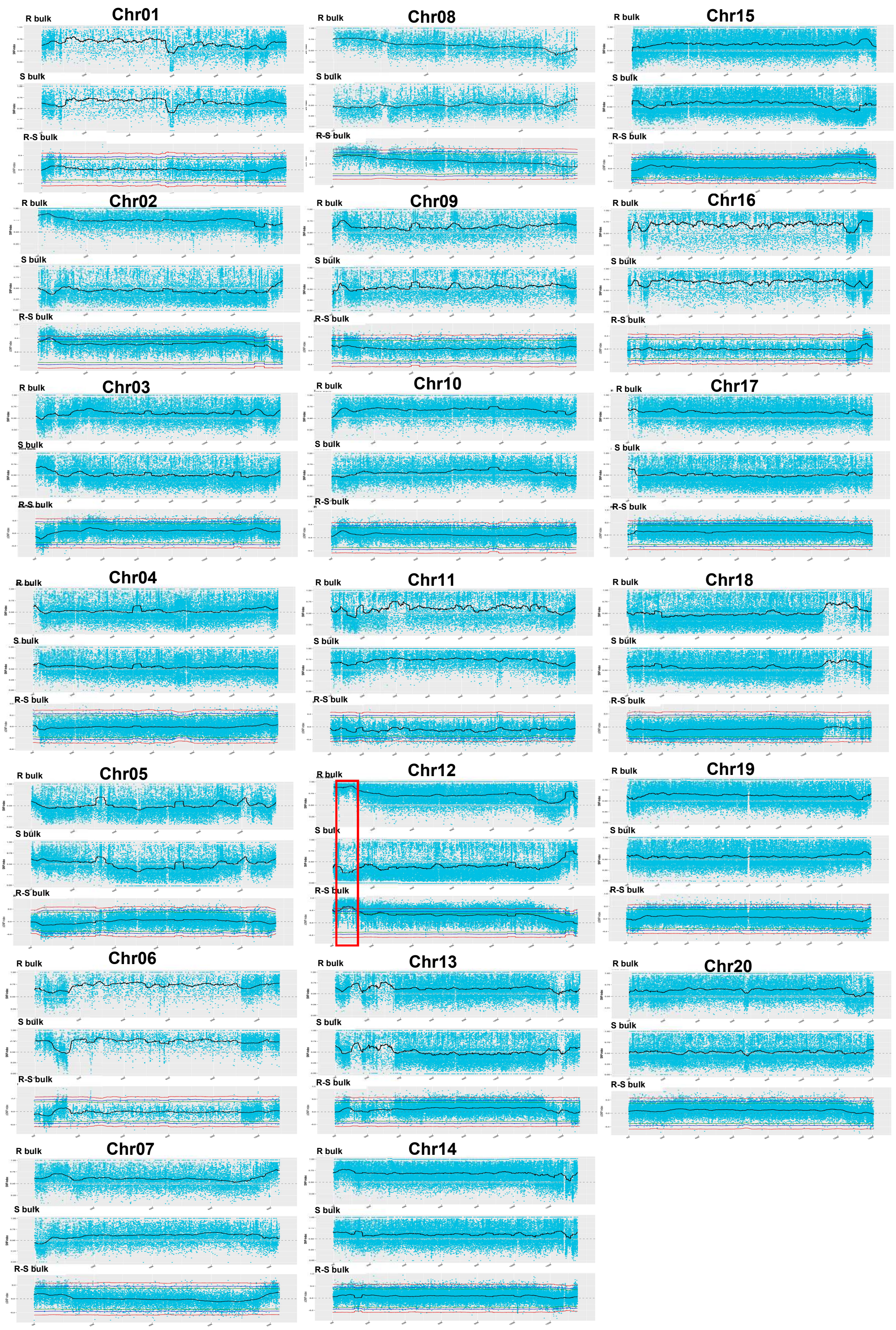

Supplementary Figure 7 SNP-index of R and S bulks and *Δ*(SNP-index) plots generated by sliding-window analysis on 20 chromosomes. The X-axis shows physical positions of chromosomes and the Y-axis the average SNP-index in each 2-Mb physical interval with a 10-kb sliding window. The red line represents the threshold line with confidence of 0.99, the blue line represents the threshold line with confidence of 0.95, and the green line represents the threshold line with confidence of 0.90.

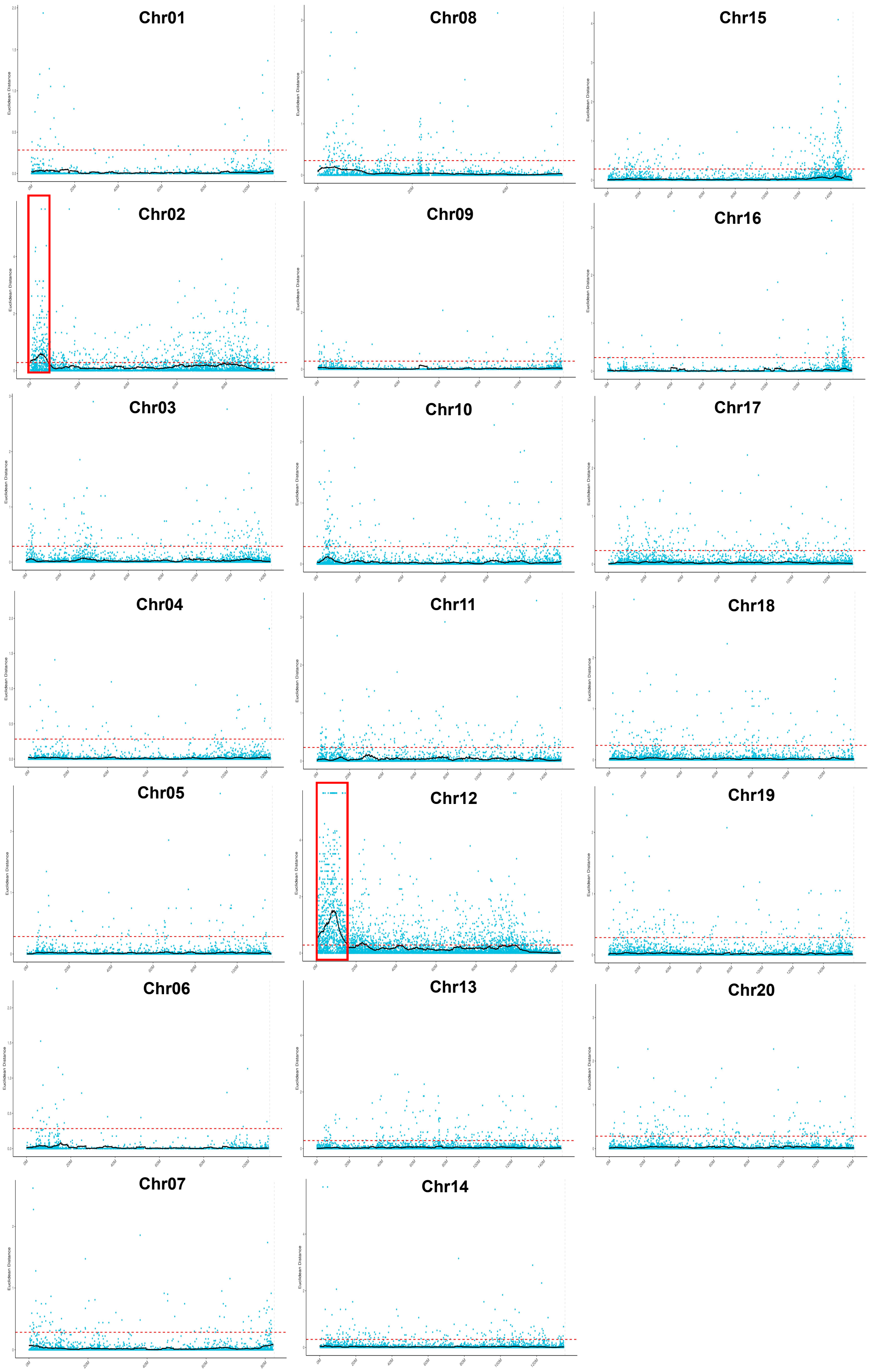

Supplementary Figure 8 Euclidean distance (ED) value distribution of InDels for different 20 chromosomes for RRSI. Red lines indicate the sliding window average of 2 Mb interval with 100 kb increment for ED value.

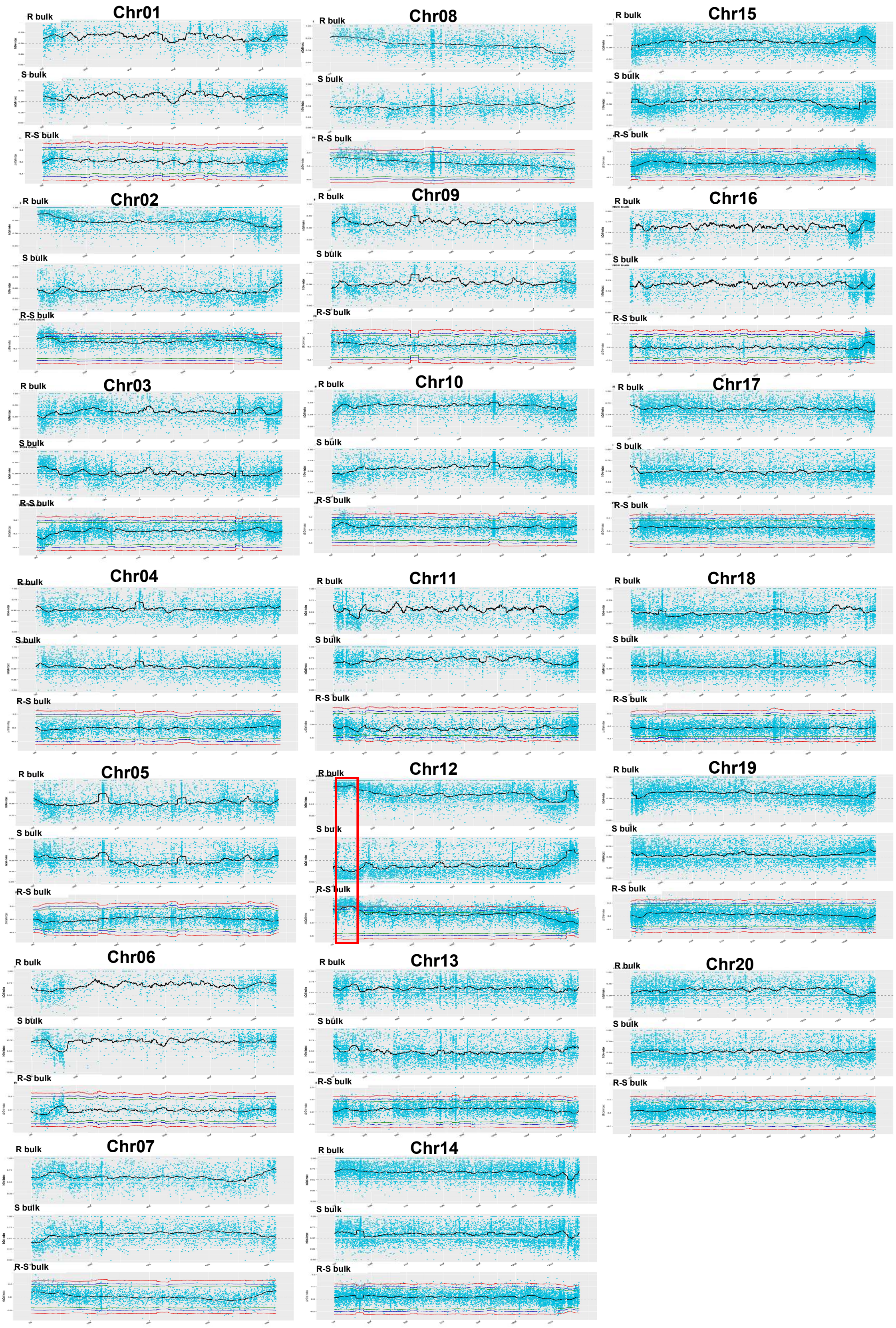

Supplementary Figure 9 InDel-index of R and S bulks and *Δ*(InDel-index) plots generated by sliding-window analysis on 20 chromosomes. The X-axis shows physical positions of chromosomes and the Y-axis the average InDel-index in each 2-Mb physical interval with a 10-kb sliding window. The red line represents the threshold line with confidence of 0.99, the blue line represents the threshold line with confidence of 0.95, and the green line represents the threshold line with confidence of 0.90.

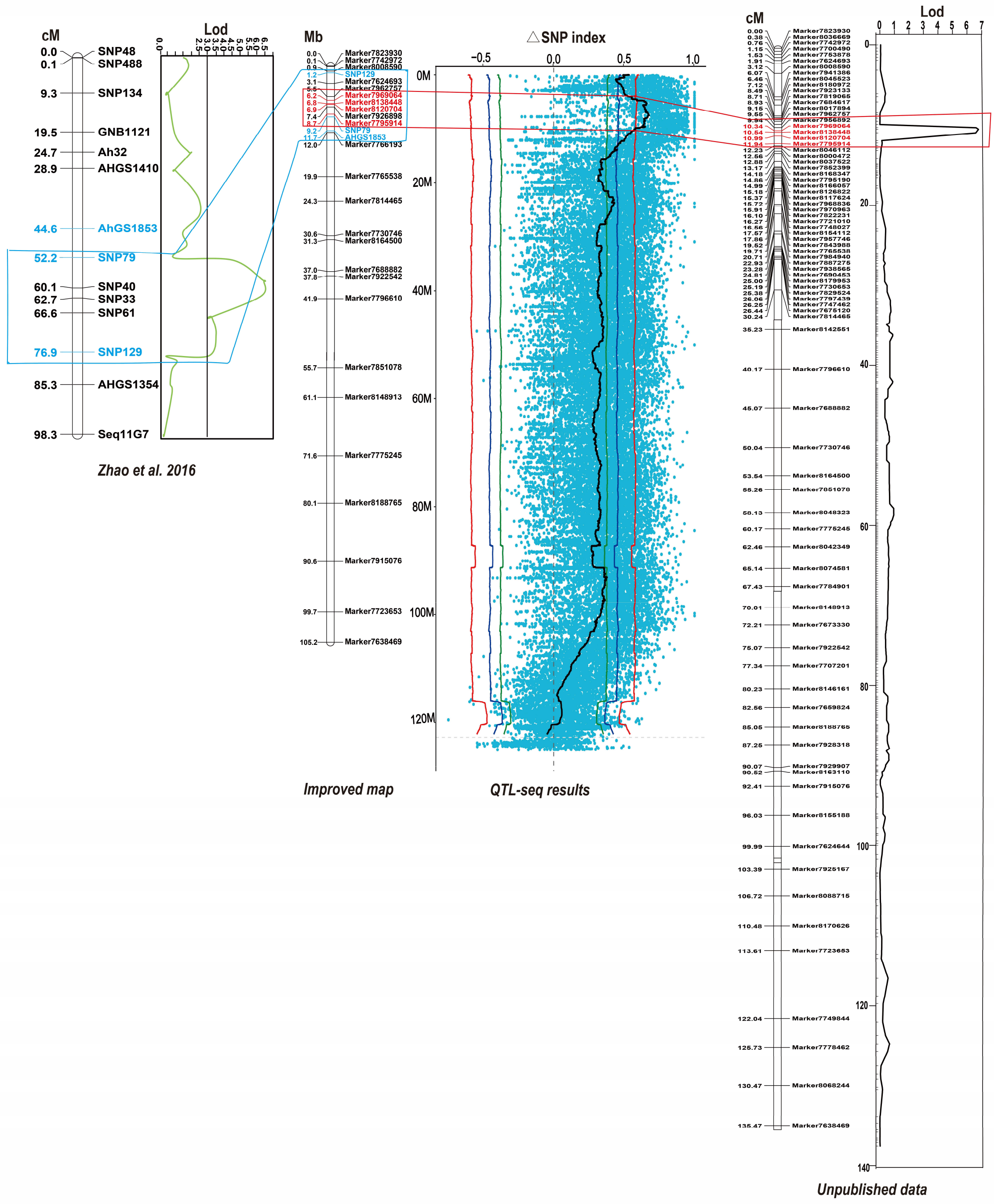

Supplementary Figure 10 Integration of newly identified diagnostic markers on genetic map and estimation of QTL effects.
